# Zika virus infections of human stem cell-derived cerebral organoids reveal viral lineage-specific pathogenesis responses

**DOI:** 10.1128/mbio.00863-26

**Published:** 2026-06-15

**Authors:** Alfred T. Harding, Yichen Zhang, Jane-Jane Chen, Jenna M. Antonucci, Alexsia Richards, Valerie Leger, Charles A. Whittaker, Yann S. Vanrobaeys, Divyansh Agarwal, Tenzin Lungjangwa, Rudolf Jaenisch, Lee Gehrke

**Affiliations:** 1 Massachusetts Institute of Technology 2167, Cambridge, Massachusetts, USA; 2 Whitehead Institute for Biomedical Research 2187, Cambridge, Massachusetts, USA; 3 Bioinformatics & Computing Core Facility of the Swanson Biotechnology Center, Koch Institute for Integrative Cancer Research, Massachusetts Institute of Technology 67320, Cambridge, Massachusetts, USA; 4 Massachusetts General Hospital 2348, Boston, Massachusetts, USA; 5 Harvard Medical School 1811 , Boston, Massachusetts, USA; Duke University School of Medicine, Durham, North Carolina, USA

**Keywords:** virus, organoid, pathogenesis, Zika, flavivirus, stress response, tolerance

## Abstract

Zika virus (ZIKV) has multiple lineages and strains that cause a range of disease severity, underscoring the need to elucidate differential neuropathogenesis mechanisms. Here, we performed systematic, side-by-side comparisons of African, Asian, and American ZIKV lineage infections using cerebral organoids derived from human embryonic stem cells, a relevant human model experimental system. African lineage ZIKV strains, as well as the ancestral Asian Malaysia strain, persistently infected neural progenitor cells, causing apoptosis and severe disruption of ventricular cytoarchitecture. In contrast, contemporary Asian and American lineage viruses were cleared from ventricles, coinciding with low apoptosis and reduced neuropathology. Single-cell RNA sequencing demonstrated upregulated cell-type-specific antiviral signaling during American lineage infections, coinciding with viral clearance from ventricular progenitor cells. Conversely, pathogenic African lineage infections were associated with apoptosis, reduced STAT2 and IFIT1 protein levels, and enhanced activation of stress pathways. African lineage and ancestral Malaysian strain infections induced mitochondrial oxidative stress. Scavenging the reactive oxygen species improved ventricular cytoarchitecture and progenitor survival, but without reducing viral titers. Together, these findings suggest that lineage- and strain-specific host stress responses, rather than viral burden alone, contribute to ZIKV-induced neurodevelopmental damage. An implication of this study is that host-directed therapeutic strategies, used to improve host tolerance to viral infection, may benefit clinical outcomes.

**IMPORTANCE:**

This study provides a systematic comparison of Zika virus (ZIKV) lineage infections in a relevant human model system to correlate mechanisms of host cellular responses with neuropathogenesis. By analyzing African, Asian, and American ZIKV infections side by side in cerebral organoids derived from human embryonic stem cells, we link persistent infection of neural progenitor cells to elevated cellular stress responses and structural disruption of organoid ventricles. Structural disruption was reduced by adding a hydroxyl radical scavenger, but without lowering viral titer. These data are important in strongly suggesting that host responses to viral infection can be of equal or greater importance than viral burden in determining pathogenesis. This study provides mechanistic insight into how closely related viral lineage infections result in divergent outcomes in the developing brain. The data have added importance for suggesting the potential value of host-directed therapeutics to improve ZIKV tolerance without addressing viral titer.

## INTRODUCTION

Zika virus is a mosquito-transmitted positive-sense single-stranded RNA virus and a member of the virus family *Flaviviridae*. Two Zika virus (ZIKV) lineages (African and Asian) were described in 2012 (1), with a third lineage (American) included in 2019 (2, 3). Fewer than 20 ZIKV infections were recorded prior to 2007 (4), when outbreaks occurred in the Yap Islands and French Polynesia (5) with Guillain-Barré syndrome occurrence (6). In 2015, an epidemic of infections was reported in the Americas, especially in northeastern Brazil (7), along with reports of fetal microcephaly and adult Guillain-Barré syndrome (5, 8–10). Zika virus is unusual among flaviviruses in its ability to cross placental barriers (11–13) and cause the teratogenic outcomes described as congenital Zika syndrome.

Viral pathogenesis is a complex, multifaceted process that pits viral RNA replication against host antiviral responses. Host cells have effective antiviral responses that degrade cellular and viral RNA (OAS-RNaseL) (14), inhibit protein synthesis (eIF2α kinases) (15), inhibit the viral RNA-dependent RNA polymerase (MX proteins) (16), or block viral RNA translation (IFIT proteins) (17). To counter these host responses, viruses can disrupt viral RNA-sensing pathways (18, 19), block interferon production (20, 21), or prevent apoptosis (22). In the face of a viral infection, host cells activate stress responses that function to maintain homeostasis and facilitate recovery from a viral attack. Stress responses have been correlated with ZIKV infections (23–25), but side-by-side comparisons of pathogenesis caused by different lineages have not been reported.

Viral protein amino acid substitutions that preceded ZIKV spread from Africa and Asia to the Americas were initially proposed to be causal in the context of enhanced ZIKV transmission, pathogenesis, or microcephaly (26–28). A prM protein S139N substitution was correlated with microcephaly in a mouse model (26), but recent experimentation has not corroborated the findings (28, 29). It has been reported that during spread into the Americas, the Brazilian ZIKV strains acquired nucleotide substitutions that increased viral virulence (30, 31); however, others found that African lineages were more pathogenic in both cell culture and murine models (12, 32, 33). In general, agreement across the field on ZIKV pathogenesis has been limited by attempts to compare or extrapolate data from different model systems, including transformed cell lines, stem cell-derived cells, rodents, and non-human primates, as well as experimentation that does not include all lineages.

We reasoned that a side-by-side comparison of ZIKV infections, using a relevant human model system, would inform an understanding of ZIKV pathogenesis and disease mechanisms. Cerebral organoids derived from human pluripotent stem cells recapitulate gene expression patterns and brain morphology during fetal development (34–37), and have been shown to be an excellent model for studying viral neuropathogenesis (36, 38, 39). Here, we have systematically infected cerebral organoids derived from human embryonic stem cells with a panel of ZIKV lineages and strains collected from several geographic locations, mirroring ZIKV transmission from Africa to Asia and the Americas. Organoid size measurements, confocal immunofluorescence imaging, RNA sequencing, protein immunoblotting, and biochemical assay data strongly suggest that pathogenesis mechanisms among ZIKV lineages include differential antiviral interferon and stress responses, mediated in part by reactive oxygen species (ROS) imbalance. Adding a hydroxyl radical scavenger chemical antioxidant improved organoid tolerance to severe ZIKV infection, suggesting the potential clinical value of host-directed therapeutics.

## RESULTS

**Fig 1:**
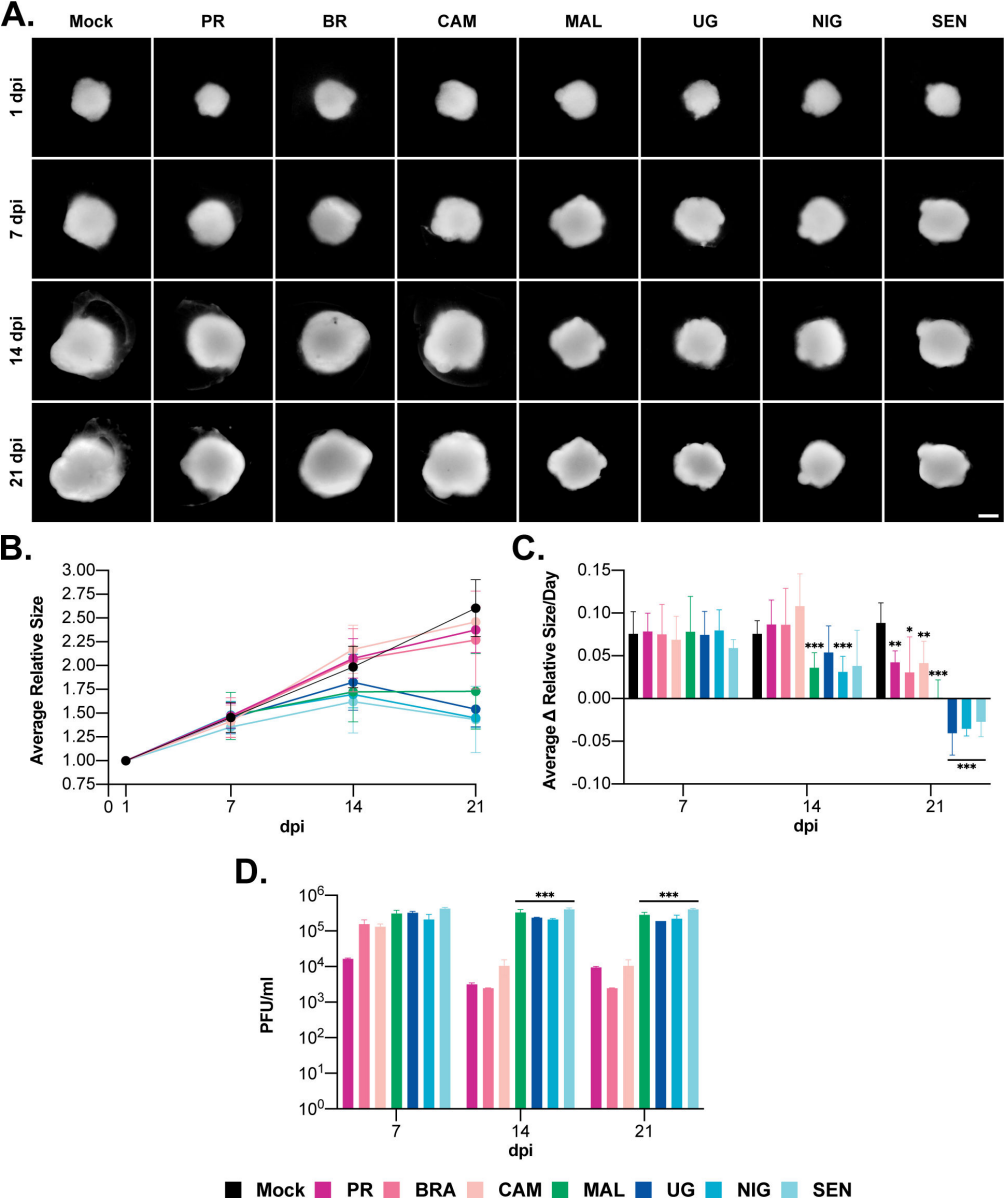
Growth characteristics of cerebral organoids infected with ZIKVs. (**A**) Darkfield microscope images of organoids at 1, 7, 14, and 21 dpi (scale bar = 500 µm). (**B**) Average size of organoids in each infection group relative to 1 dpi at 7, 14, and 21 dpi (*n* = 9 organoids; mean ±  SD). (**C**) Average change in size/day at 7, 14, and 21 dpi of organoids in each infection group (*n* = 9 organoids, *P ≤ 0.05, **P  <  0.01, ***P <  0.001 compared to Mock; mean  ±  SD). (**D**) Viral titers representing 24 h of released virus at 7, 14, and 21 dpi (*n* = two independent experiments, each with triplicate groups of three organoids that were infected and maintained in separate wells before being titered in independent plaque assays. ***P <  0.001 compared to PR, BR, and CAM; mean  ±  SD).

Seven ZIKV strains spanning Asian (Malaysia [MAL] and Cambodia [CAM]), American (Puerto Rico [PR] and Brazil [BR]), and African (Senegal [SEN], Nigeria [NIG], Uganda [UG]) lineages were studied (Fig. S1A). Cerebral organoids generated from day 21 post-embryoid body formation recapitulated first-trimester human brain development (Fig. S1B) (40). Asian/American lineage infections did not impair organoid growth significantly, whereas African lineage strains (ZIKV-UG, -SEN, -NIG) caused growth arrest by 14 dpi (Fig. 1A through C). Although classified as Asian lineage, ancestral ZIKV-MAL infections showed a growth phenotype similar to African strains (Fig. 1B). Plaque assays revealed that ZIKV-PR produced 10–15-fold fewer infectious virus particles (plaque forming units, PFU) at 7 dpi compared to other strains (Fig. 1D). By 14 and 21 dpi, PFU levels from Asian/American infections declined ~2 logs relative to ZIKV-MAL and African strains, which remained constant. These results suggest that replication and spread of the Asian/American ZIKV were controlled by host anti-viral responses, while replication and spread of African lineage viruses and ZIKV-MAL were unimpeded.

### ZIKV-induced growth defect severity correlates with cytoarchitectural disruption

**Fig 2:**
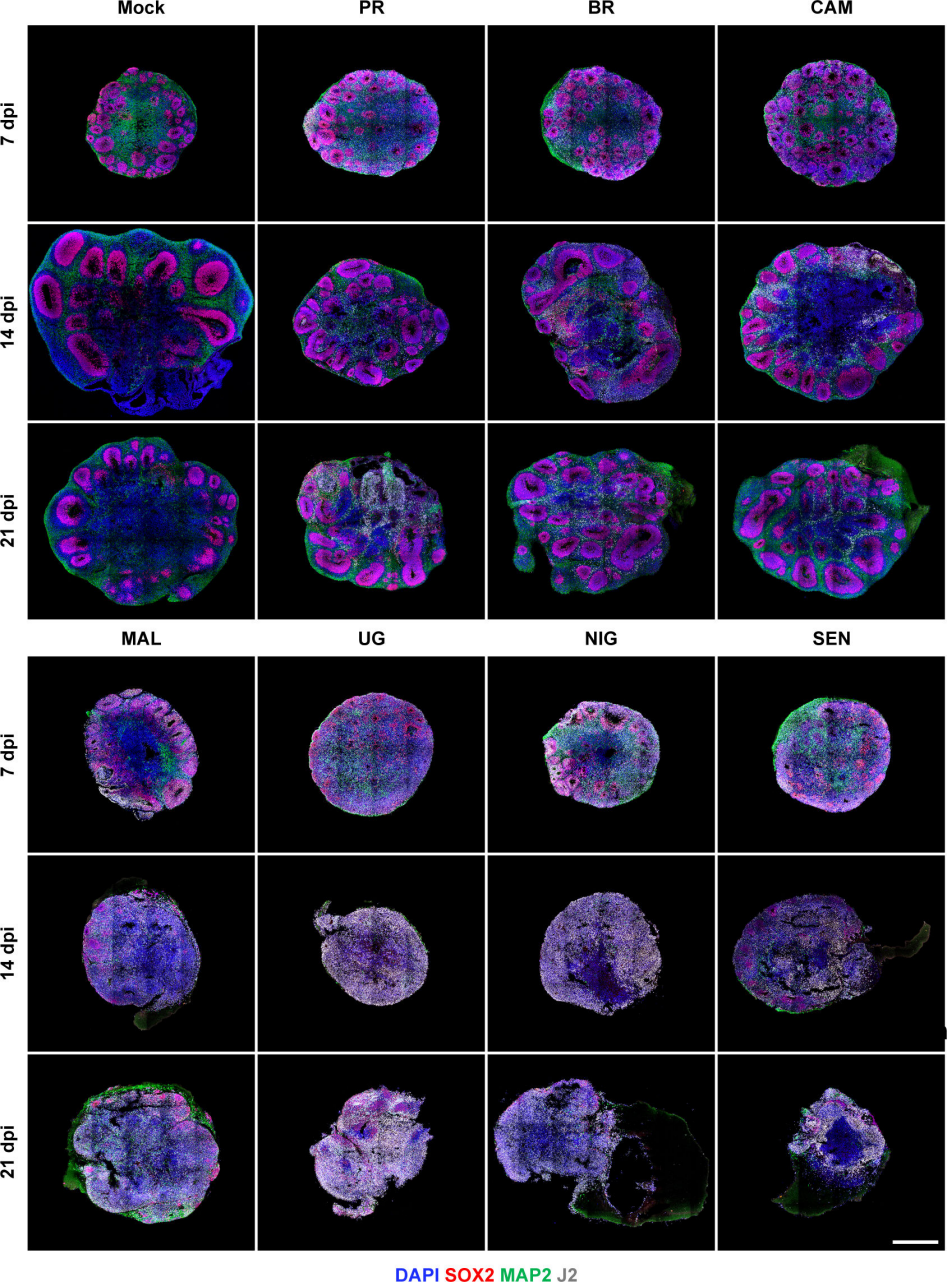
Confocal imaging of cerebral organoids infected by seven ZIKV strains at 7, 14, and 21 dpi. Anti-SOX2 antibody (red, progenitor cells), anti-MAP_2 antibody (green, neuron somata, and dendrites), and double-stranded viral replicative form RNA antibody (white, J2), and DAPI (blue). Scale bar: 500 μm.

Organoid ventricles are located near the periphery of organoid sections and are analogous to human brain ventricles. An organoid schematic (Fig. S2) shows that ventricles are composed of neuronal progenitor cells (NPC), intermediate progenitor cells (IPC), and radial glia, collectively referred to here as ventricular cells. Neurons, glia, astrocytes, and oligodendrocytes are described as surrounding the organized ventricles (41). Immunofluorescence imaging of organoid sections was done using anti-SOX2, anti-MAP2, and anti-dsRNA (J2) antibodies. In mock and Asian/American infections at 7 dpi, the SOX2+ ventricles are intact and well-organized and surrounded by MAP2+ neurons (Fig. 2; Fig. S2) (40). At 14 dpi, the ventricle sizes increased in the mock-infected organoids, while the total number of ventricles decreased. These ventricles were maintained through 21 dpi, along with the surrounding interlaced meshwork of MAP2+ neurons. Conversely, at 14 dpi, organoids infected by ZIKV-MAL or African lineage ZIKV showed cytoarchitectural disorganization and diminished SOX2 staining, suggesting loss of neuronal progenitor cells (42) and the neuropil (dense meshwork of axons and dendrites) at 14 and 21 dpi. The immunofluorescence data suggest that the organoid growth defects (Fig. 1) correlate with loss of SOX2+ cells and disruption of the MAP2+ organoid neuropil and cytoarchitecture.

### Viral RNA clearance differs by lineage

**Fig 3:**
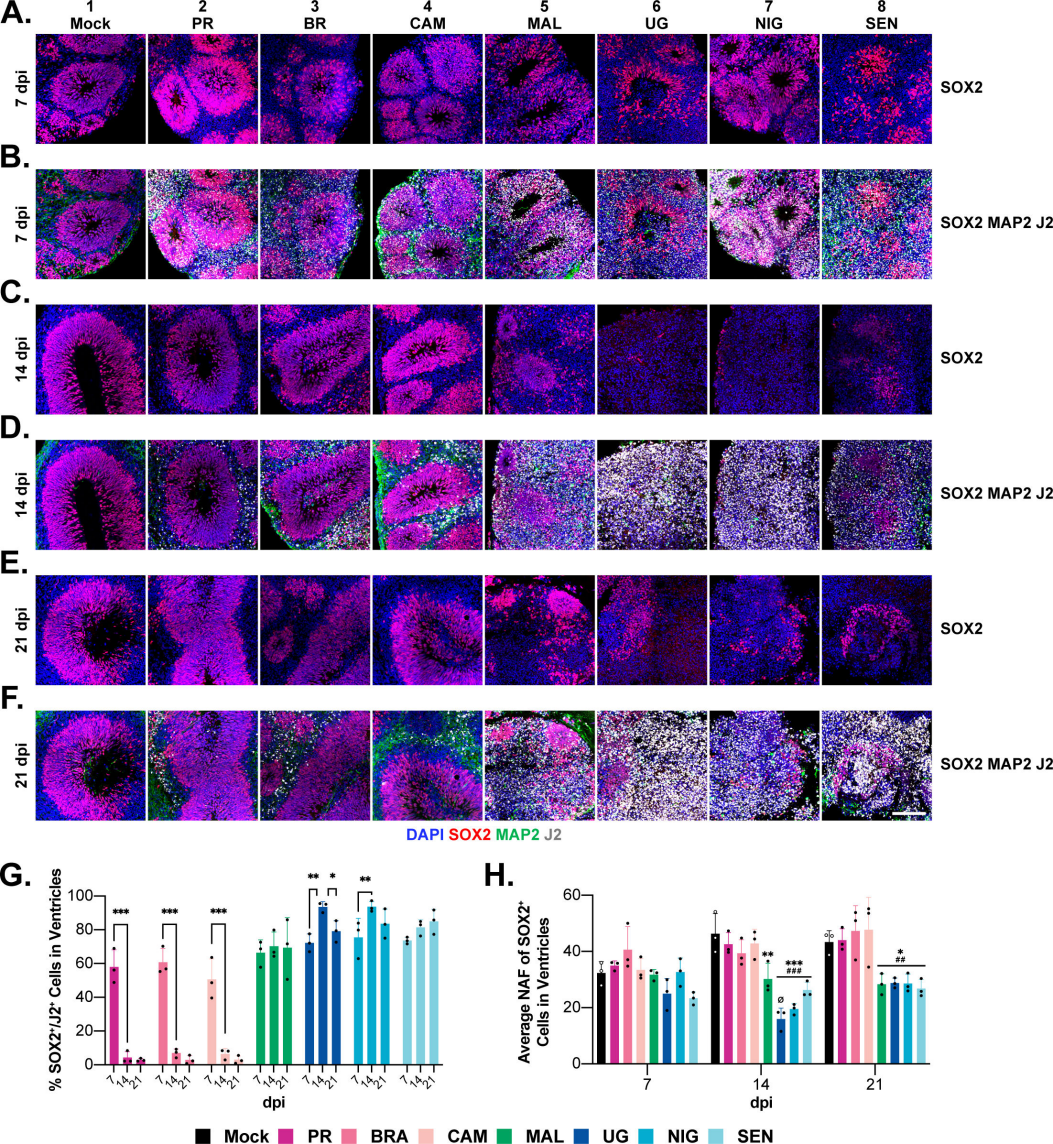
Immunofluorescence microscopy visualizes ZIKV infection persistence in SOX2-positive cells. Confocal microscopy images showing ventricle regions of human cerebral organoids that were mock-infected or infected with ZIKV and then sectioned and stained with anti-SOX2 antibody (red, progenitor cells), anti-MAP2 antibody (green, neuron somata, and dendrites), anti-double-stranded viral replicative RNA antibody (white, J2), and DAPI (blue) at 7, 14, and 21 dpi (scale bar = 100 µm). For panels **G–H**, the statistical annotation is *n* = 3 independent fields of view, *P ≤ 0.05, **P  <  0.01, ***P <  0.001, * compared to Mock, ^#^ compared to PR, BR, and CAM, ^Ø^ compared to MAL; mean  ±  SD. (**A**) 7 dpi SOX2/DAPI. (**B**) 7 dpi SOX2/MAP2/J2/DAPI. (**C**) 14 dpi SOX2/DAPI. (**D**) 14 dpi SOX2/MAP2/J2/DAPI. (**E**) 21 dpi SOX2/DAPI. (**F**) 21 dpi SOX2/MAP2/J2/DAPI. (**G**) Percentages of ventricular cells positive for SOX2 and J2 staining at each time point, normalized to the number of SOX2+ cells. (**H**) Average nuclear area factor (NAF) at each time point.

**Fig 4:**
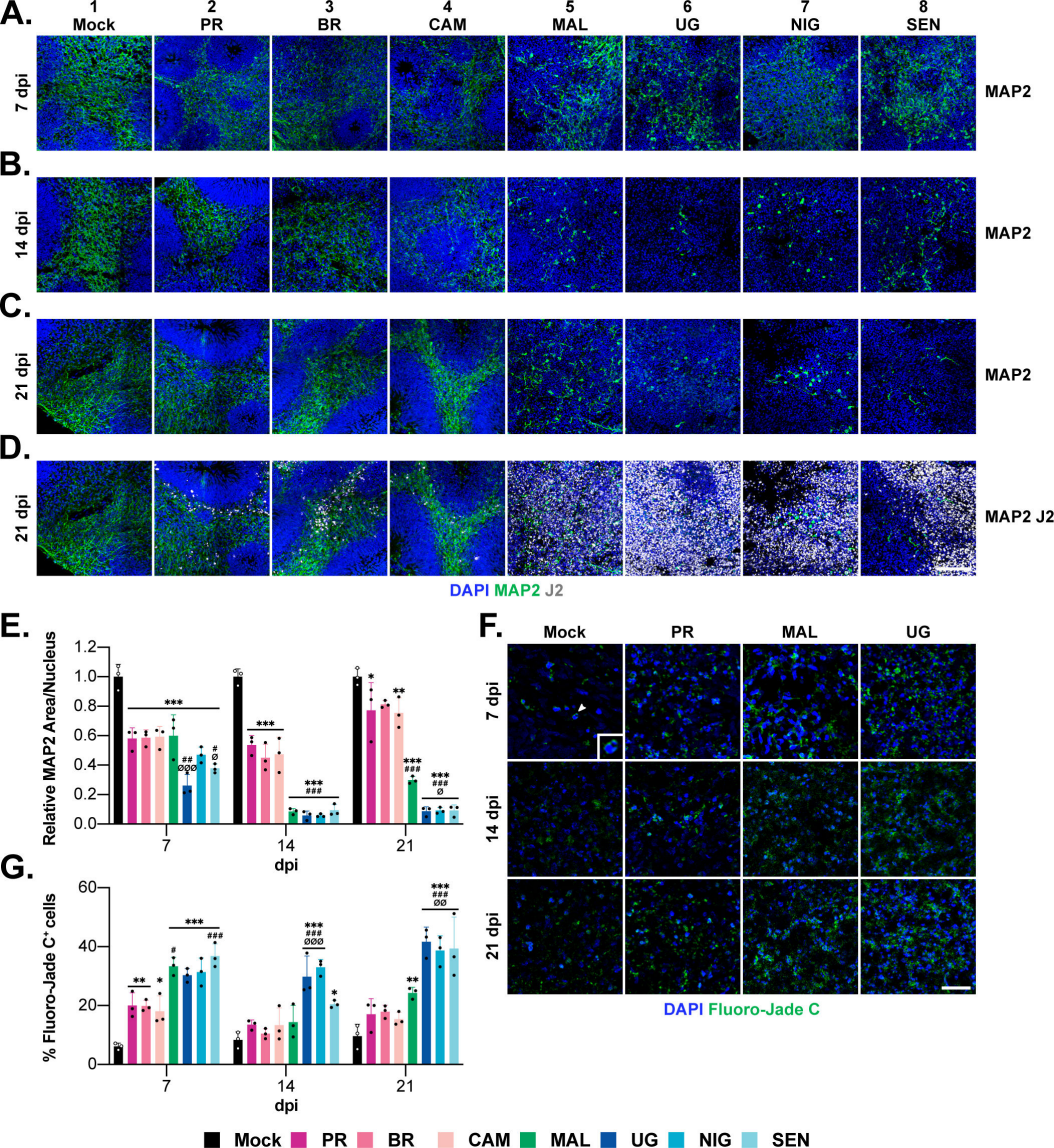
Immunofluorescence microscopy visualizes ZIKV infection in neurons. Confocal microscopy images showing MAP2+ neurons and other cells in the extraventricular regions of human cerebral organoids, stained with anti-MAP2 antibody (green, neuron somata, and dendrites), anti-double-stranded viral replicative RNA antibody (white, J2), and DAPI (blue) at 7, 14, and 21 dpi (scale bar = 100 µm). (**A–C**) 7, 14, and 21 dpi MAP2/DAPI. (**D**) 21 dpi MAP2/J2/DAPI. Note that the J2 signal in columns 5–8 includes neurons and NPCs. (**E**) Average relative MAP2 area at each time point (*n* = 3, *P ≤ 0.05, **P  <  0.01, ***P  <  0.001, * compared to Mock, ^#^ compared to PR, BR, and CAM, ^Ø^ compared to MAL; mean  ±  SD). (**F**) Confocal microscopy images of extraventricular regions stained with Fluoro-Jade C (FJC) (green) at 7, 14, and 21 dpi in Mock, ZIKV-PR, ZIKV-MAL, and ZIKV-UG infections. The arrow in Mock 7 dpi depicts an FJC+ cell with a zoomed-in version in the lower right corner (scale bar = 25 µm). (**G**) Percentages of FJC+ cells at each time point (*n* = 3, *P ≤ 0.05, **P < 0.01, ***P < 0.001, * compared to Mock, ^#^ compared to PR, BRA, and CAM, ^Ø^ compared to MAL; mean  ±  SD).

Organoid sections were viewed at higher magnification to examine cytoarchitecture and quantify virus-infected SOX2+ ventricle cells (Fig. 3). SOX2-only staining is shown in Fig. 3 (rows A, C, E) while the corresponding J2 channel images are presented in Fig. 3 (rows B, D, F). Co-staining with anti-SOX2 and anti-double-stranded RNA (J2) antibodies revealed infected progenitor cells (Fig. 3G). When organoids were infected by Asian/American lineage ZIKV, 60% of SOX2+ cells were infected at 7 dpi (Fig. 3, row B, columns 2–4; Fig. 3G); however, by 14 dpi, there was a precipitous decrease in the percentage of ventricular SOX2+/J2+ cells, which remained low through 21 dpi (Fig. 3, rows B, columns 2–4; Fig. 3G). J2 signal was also diminished in the extraventricular areas that are expected to be occupied by mature neurons, astrocytes, and oligodendrocytes (Fig. 3, rows D and F, columns 2–4); however, the decrease was not as pronounced as in the ventricles. The J2 signal persisted in ventricular progenitor cells infected by ZIKV-MAL and the African ZIKV strains, remaining at 70%–90% infected over the time course (Fig. 3, rows B, D, and F, columns 5–8; Fig. 4G). Colocalization analyses confirmed that the dsRNA signal emanated from cells expressing the viral envelope protein (Fig. S3). The results (Fig. 3) suggest that host cells blunted the replication and spread of Asian/American ZIKV lineage viruses, resulting in virus clearance; however, the ZIKV-MAL and the African lineage ZIKV infections were persistent.

### African lineage infections cause apoptosis

ZIKV infection has been shown to cause NPC apoptosis (42–46). Because nuclear morphology has been extensively studied as an indicator of apoptosis (47, 48), we assessed apoptosis by quantifying nuclear area factor (NAF) values. NAF is calculated as the nuclear area divided by nucleus circularity (49, 50), wherein high NAF levels correlate with healthy cells, and low NAF values correlate with very circular nuclei and apoptosis/cell death. At 7 dpi, the NAF values of the seven ZIKV strain infections were not significantly different, although ZIKV-UG and ZIKV-SEN-infected organoids did have smaller average nuclear areas (Fig. 3H). However, at both 14 and 21 dpi, ZIKV-MAL and African lineage ZIKV infections had significantly lower comparative NAF values, suggesting differential apoptosis responses. The NAF data (Fig. 3H), as well as organoid cleaved caspase staining (Fig. S4), suggest that apoptotic cell death correlates with differential organoid pathogenesis across the three ZIKV lineages (Fig. 3A through F).

### Neuronal damage is ZIKV lineage-dependent

Damage to neuronal cell bodies (somata) and dendrites was evaluated using anti-MAP2 and Fluoro-Jade C imaging (Fig. 4). The neuropil was disrupted at 7 dpi in all ZIKV infections (Fig. 4A); moreover, the MAP2 signal was correspondingly reduced (Fig. 4E). At 21 dpi, dsRNA immunoreactivity was nearly absent from the SOX2+ ventricles of organoids infected by ZIKV-PR, -BR, and -CAM (Fig. 3, rows A–E and columns 1–4), but persisted in extra-ventricular cells (Fig. 4, row D, columns 2–4). Disrupted neuropil was observed at 7 dpi of the ZIKV-PR, -BR, and -CAM infections, but did not progress through 14 and 21 dpi (Fig. 4, rows B–C, panel E). ZIKV-MAL and African lineage virus infections disrupted the neuropil cytoarchitecture (Fig. 4, rows A–C and columns 5–8), and the corresponding MAP2 area/nucleus decreased precipitously at the 14 and 21 dpi time points (Fig. 4E). These data suggest that Asian/American ZIKV replication and spread were checked by host antiviral responses, while ZIKV-MAL and African lineage virus infections were unchecked, permitting persistent virus replication, spread, and cytoarchitecture disruption. Suspected neuronal degeneration (Fig. 4 row D, columns 5–8) was confirmed by Fluoro-Jade C (FJC) staining (51) (Fig. 4F; quantified in Fig. 4G). These data strongly suggest that ZIKV-UG infection caused neuronal damage.

### Bulk RNA-seq reveals differential stress responses among ZIKV lineage infections

**Fig 5:**
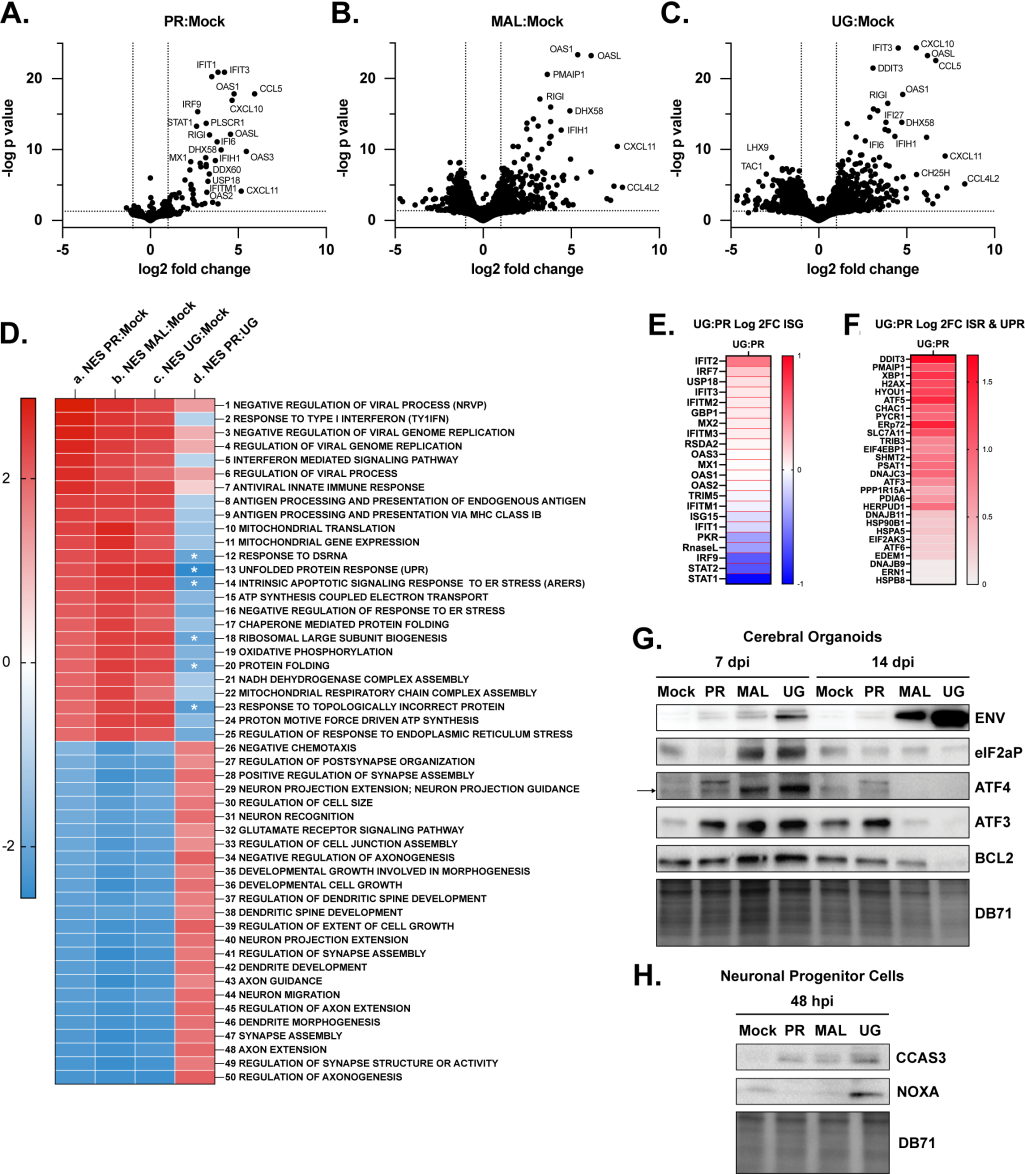
Bulk RNA-seq analysis of cerebral organoids infected with ZIKV-PR, ZIKV-MAL, and ZIKV-UG. (**A–C**) Volcano plots showing differentially expressed genes using cutoffs of adjusted *P* ≤ 0.05 and absolute fold-change ≥ 2. (**D**) Top 25 positively enriched GSEA pathways (1–25) and the top 25 negatively enriched GSEA pathways (26–50). (Column a) PR:Mock NES comparison; (column b) MAL:Mock NES comparison; (column c) UG:Mock NES comparison; (column d) PR:UG NES value comparison. The titles of the GSEA pathways are shown at the right of panel **D**. All NES values in columns a–c are statistically significant at *P-*adj ≤ 0.05. Cells indicated by an asterisk (*) in column d are statistically significant at *P-*adj ≤ 0.05. (**E**) Heatmap showing log2-FCs in selected ISR/UPR genes in a UG:PR comparison. Log2-FC values are significant at *P* ≤ 0.01 for DDIT3 to eIF4EBP. (**F**) Log2-fold gene expression differences for selected ISGs in a UG:PR comparison. (**G**). Immunoblot showing protein levels of unfolded protein response (UPR) and integrated stress response (ISR) proteins using 7 and 14 dpi organoids infected by ZIKV-PR, ZIKV-MAL, or ZIKV-UG. ENV: viral envelope protein; DB-71: direct blue protein stain. The arrow in the ATF4 panel shows the migration of the ATF4 protein. (**H**) Immunoblot showing cleaved caspase 3 (CCAS3) and NOXA protein levels in NPC infected by ZIKV-PR, ZIKV-MAL, and ZIKV-UG. The immunoblots are representative of two independent experiments with one technical replicate that sampled three organoids at each time point.

Bulk RNA-seq revealed increases in the differentially expressed genes as a function of viral pathogenesis severity, PR < MAL < UG (Fig. 5A through C). Interferon (IFN) and interferon-stimulated gene (ISG) expression are hallmarks of virus infections (52), and indeed, two interferon gene set enrichment analysis (GSEA) pathways were among the top positively enriched (Fig. 5D, rows 2, 5). In virus:mock comparisons, the top seven GSEA pathways are positively perturbed and antiviral (Fig. 5D, rows 1–7), including ISGs; however, none of the enrichment scores reached statistical significance (*P-*adj ≤ 0.05) in the PR:UG NES comparison (Fig. 5D, column d). To further probe the antiviral pathways, log2-fold gene expression values (log2-FC) were examined (Fig. 5E). Interestingly, none of the ISG log2-fold values was upregulated with statistical significance (*P-*adj ≤ 0.05) in ZIKV-UG as compared to ZIKV-PR, whereas the major regulators of IFN signaling, IRF9 and STAT1/2, were downregulated, with only STAT1 reaching statistical significance (*P-*adj ≤ 0.05). The combination of NES perturbation and log2-FC data suggests that antiviral interferons and ISGs were not strongly upregulated in severe ZIKV-UG infections as compared to ZIKV-PR. These data suggest that signaling to ISG expression was disrupted in the ZIKV-UG infections, consistent with other reports stating that ZIKV infections suppress interferon responses (53).

Further examination of the bulk RNAseq NES data revealed statistically significant (*P-*adj ≤ 0.05) negative perturbation of unfolded protein response (UPR) and integrated stress response (ISR) pathways in ZIKV-PR infections as compared to ZIKV-UG (Fig. 5D, column d, rows 12–14, 20, 23). The UPR overlaps in part with the ISR and is activated by the accumulation of improperly folded proteins in the endoplasmic reticulum (54), followed by eIF2α phosphorylation and host protein shutoff as a cell survival mechanism (55). To extend the bulk RNAseq analysis, we analyzed log2-FC differential gene expression for 28 UPR/ISR genes (Fig. 5F). These data demonstrate that all genes were comparatively upregulated in ZIKV-UG infections, with 12 reaching statistical significance (*P-*adj ≤ 0.05; DDIT3-eIF4EBP1). The log2-FC data are therefore consistent with the bulk RNAseq NES data (Fig. 5D) in demonstrating positive perturbation or upregulation of UPR/ISR genes by the severe ZIKV-UG infections, as compared to ZIKV-PR.

We next validated the transcriptional analysis using immunoblotting, demonstrating that, in comparing ZIKV-PR and ZIKV-UG infections, phosphorylated eF2α and ISR proteins ATF4/ATF3 show elevated signals at 7 dpi, consistent with UPR/ISR activation (Fig. 5G). In addition, caspase 3 cleavage, suggesting apoptosis, was evident in ZIKV-UG infections (Fig. S4). Anti-apoptotic BCL2 protein levels were diminished while pro-apoptotic NOXA levels were elevated in ZIKV-UG infections (Fig. 5G and H). A summary of the bulk RNAseq data is, first, that despite ZIKV-UG persistence (Fig. 3), ISG expression was not differentially upregulated in comparison to ZIKV-PR (Fig. 5E). Second, log2-FC (Fig. 5F) and immunoblotting (Fig. 5G) are evidence that the UPR and ISR were differentially upregulated in severe ZIKV-UG infections, correlating with elevated stress responses and the severe pathogenesis observed in imaging (Fig. 2 and 3).

### Single-cell RNA-seq reveals differential lineage responses

**Fig 6:**
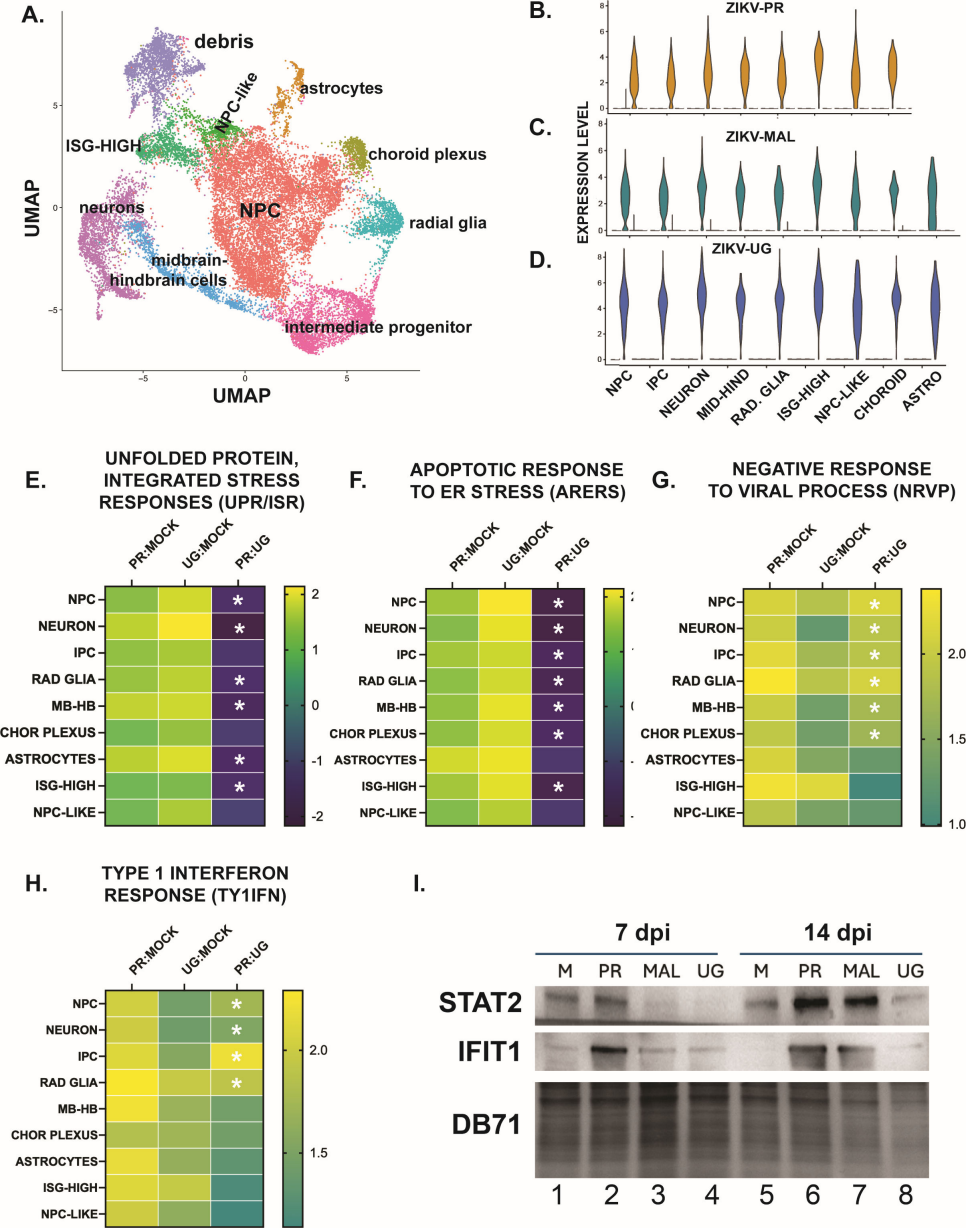
Single-cell RNA-seq reveals differential pathway enrichments that distinguish cell types and ZIKV lineages. (**A**) UMAP clustering of single-cell RNA-seq data. NPC: neuronal progenitor cell; ISG: interferon-stimulated gene. (**B–D**) Viral RNA distribution expression levels among cell types infected by ZIKV-PR, ZIKV-MAL, or ZIKV-UG. (**E**) Heatmap showing PR:MOCK, UG:MOCK, and PR:UG NES value comparisons for the Hallmark unfolded protein and integrated stress curated list of coherently expressed genes, for each cell type. The scale reference bar indicates the NES value. (**F**) Heatmap showing PR:MOCK, UG:MOCK, and PR:UG NES value comparisons for the ARERS gene ontology analysis. (**G**) Heatmap showing PR:MOCK, UG:MOCK, and PR:UG NES value comparisons for the negative response to viral process (NRVP) gene ontology analysis. (**H**) Heatmap showing PR:MOCK, UG:MOCK, and PR:UG NES value comparisons for the type 1 interferon (TY1IFN) gene ontology analysis. Asterisks (*) indicate statistical significance at *P-*adj ≤ 0.05. (**I**) Immunoblot, probing for STAT2 and IFIT1 proteins for cerebral organoids infected by ZIKV-PR, ZIKV-MAL, or ZIKV-UG, and analyzed at 7 or 14 dpi. DB-71: direct blue 71 protein stain as loading controls.

Single-cell RNA sequencing (scRNA-seq) was used to identify organoid cell types, their infection status, and their transcriptome changes following virus infections. scRNA-seq identified nine cell clusters (Fig. 6A; Fig. S5). Viral RNA levels followed a PR < MAL < UG hierarchy across most cell types (Fig. 6B through D), without evidence of differential infection tropisms. NES values were compared to assess differential gene expression. For the GSEA ISR/UPR and apoptotic response to endoplasmic reticulum stress (ARERS) pathways (Fig. 5D), NES values were positively perturbed in PR:MOCK and UG:MOCK infections (Fig. 6E and F). The virus:virus comparisons (PR:UG) demonstrated that the NES responses were negatively perturbed in ZIKV-PR in comparisons to ZIKV-UG (Fig. 6E and F), equating to positive enrichment in ZIKV-UG infections. The results are consistent with the organoid imaging (Fig. 2 and 3) in correlating elevated ISR/UPR and apoptosis stress responses with the cytoarchitecture damage observed in ZIKV-UG infections (Fig. 2 and 3).

Negative response to viral process (NRVP) and type 1 interferon (TY1IFN) pathways reflect antiviral responses. In both lineage infections, NES scores were positively perturbed across all cell types in PR:MOCK and UG:MOCK comparisons (Fig. 6G and H). To examine potential differences between PR and UG infections, PR:UG comparisons demonstrated that antiviral NRVP responses were positively perturbed to levels with statistical significance in 6/9 cell types (Fig. 6G). The TY1IFN responses were positively perturbed in NPC, IPC, and radial glia, which comprise ventricles, as well as in surrounding neurons (Fig. 6H; Fig. S2). The PR:UG comparison data strongly suggest that, at single-cell levels, antiviral and type 1 interferon pathways are upregulated in American lineage ZIKV-PR infections and comparatively downregulated in African lineage ZIKV-UG infections. We interpret the ZIKV-PR low pathogenesis and virus clearance to suggest that host antiviral responses were not impaired, permitting the establishment of the antiviral environment through NRVP and TY1IFN pathways, and limiting virus infection and spread that promote virus clearance.

It was reported previously that the key interferon response signaling protein, STAT2, is degraded in dengue and Zika virus infections (21, 56). Immunoblotting revealed detectable STAT2 protein in ZIKV-PR infections (Fig. 6I, lanes 2 and 6); however, STAT2 protein levels were diminished in the ZIKV-MAL and ZIKV-UG infections at both 7 and 14 dpi (Fig. 6I, lanes 3, 4, 7, 8). We further examined IFIT1, which is an interferon-stimulated antiviral protein and a target of JAK-STAT signaling. IFIT1 levels were detectable in ZIKV-PR infections at both 7 and 14 dpi (Fig. 6I, lanes 2 and 6), while diminished in the ZIKV-MAL and ZIKV-UG infections (lanes 3, 4, 7, 8). Diminished STAT2 and IFIT1 levels likely indicate compromised antiviral signaling in ZIKV-UG and ZIKV-MAL infections as compared to ZIKV-PR.

### Reactive oxygen species imbalance contributes to African lineage pathogenesis

**Fig 7:**
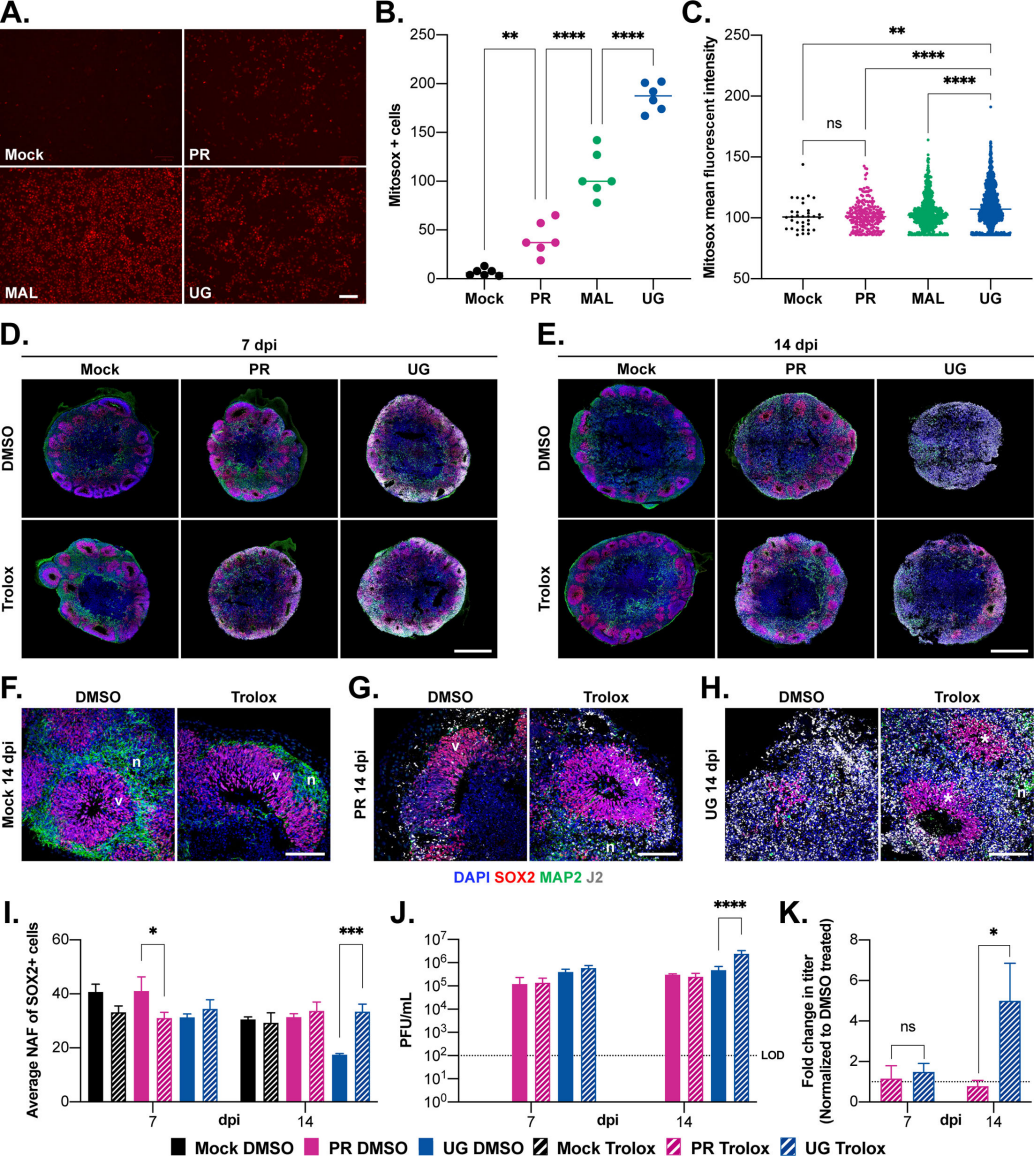
Trolox, a hydroxyl radical scavenger, partially protects ZIKV-UG-infected cerebral organoids from severe pathogenesis. (**A**) Visualization of superoxide production in ZIKV-infected Vero cells using MitoSOX Red dye (scale bar = 100 µm). (**B and C**) Quantification of ROS+ cells and ROS fluorescence intensity per cell for each of the ZIKV infections shown in panel **A**. (**D**) Confocal immunofluorescence microscopy of cerebral organoids at 7 dpi following ZIKV-PR or ZIKV-UG infection and treated with the dimethyl sulfoxide (DMSO) carrier or DMSO containing 100 μM Trolox (scale bar = 500 µm). (**E**) Same as panel **D** at 14 dpi. (**F–H**) Zoomed-in views of organoids that were mock-infected (**F**), ZIKV-PR-infected (**G**), or ZIKV-UG-infected (**H**) with the addition of either DMSO or Trolox. n: MAP2-stained neuron; v: SOX2-stained ventricle. The arrow in panel **H** shows SOX2+ cells following ZIKV-UG infection. Asterisks (*) in panel **H** mark ventricles that were visible with Trolox addition to ZIKV-UG infections (scale bar = 100 µm). (**I**) NAF analysis of images in panels **D–E** to assess apoptosis at 7 and 14 dpi, with and without Trolox treatment. (*) *P* ≤ 0.05; (***) *P* ≤ 0.001, (**J**) Viral titers representing 24 h of released virus at 7 and 14 dpi in Trolox-treated and control organoids. LOD: limit of detection. (**K**) Fold change in viral titers at 7 and 14 dpi following resulting from Trolox addition. (****) *P* ≤ 0.0001. ns: not significant.

Stress responses in viral infection often correlate with ROS imbalance associated with mitochondrial dysfunction (57–60). MITOSOX fluorescence showed that mitochondrial superoxide levels were comparatively elevated in ZIKV-MAL and ZIKV-UG-infected Vero cells, as compared to ZIKV-PR (Fig. 7A through C). To test the impact of neutralizing ROS effects on organoid cytoarchitecture, Trolox, a vitamin E superoxide scavenger, was added to organoid cultures. While Trolox effects were minimal at 7 dpi (Fig. 7D), there was a clear impact on 14 dpi ZIKV-UG-infected organoids. Instead of severe pathogenesis (Fig. 3), we observed organized ventricles, SOX2+ cells, and MAP2+ neurons (Fig. 7E through H). Apoptosis was determined by NAF analysis. Trolox caused a slight pro-apoptotic effect (lower NAF values) in ZIKV-PR-infected organoids at 7 dpi (Fig. 7I). This effect was unexpected, although Trolox has been reported to induce pro-apoptotic activity under certain conditions (61). Increased NAF values, indicating reduced apoptosis, were observed with Trolox in ZIKV-UG-infected organoids at 14 dpi (Fig. 7I), suggesting that ROS-induced apoptosis disrupts cytoarchitecture and loss of SOX2+ cells in African lineage infections. Pathogenesis reduction by Trolox could be explained by diminished apoptosis or by impeding virus replication and spread. Plaque assays showed that at 14 dpi, Trolox addition correlated with a fivefold increase in viral titer (Fig. 7J through K). These data suggest that the improved cytoarchitecture is caused by reducing ROS-induced apoptosis, and that ZIKV-UG viral titer is not wholly causal for ZIKV pathogenesis.

## DISCUSSION

ZIKV remains a pathogen of pandemic potential, yet the mechanisms underlying lineage-specific pathogenesis are incompletely understood. Using cerebral organoids derived from embryonic stem cells, we experimentally compared African, ancestral Asian (ZIKV-MAL), and contemporary Asian/American ZIKV lineage infections in cerebral organoids, which preserve intrinsic innate immune signaling (62, 63). Mechanistic insight came from imaging studies, revealing that Asian/American ZIKV was cleared from organoid ventricles, while persisting in African lineage infections. Our interpretation, supported by STAT2 and IFIT1 immunoblots (Fig. 6I), is that ZIKV-PR, an Asian/American ZIKV, has low or delayed antagonism of host antiviral and interferon responses, while ZIKV-MAL and African lineage ZIKV rapidly antagonize antiviral responses by reducing STAT2 and other ISG protein levels, leading to persistent viral replication and subsequent UPR/ISR activation (21, 64). The results (Fig. 5) strongly suggest that ISR hyperactivation in severe ZIKV-UG infections activated DDIT3-NOXA-mediated apoptosis (65), while conversely, moderate ISR activation in ZIKV-PR infections was adaptive and promoted cell survival.

Although ZIKV-MAL is classified within the Asian lineage, its pathogenesis was intermediate between other Asian/American and African ZIKV strains. ZIKV-MAL diverged early from African lineages (1). Compared with African viruses, strains derived from P6-740 Malaysia carry only 26 amino acid substitutions in the polyprotein, highlighting their similarity to African ZIKV (66). It is not clear how these mutations contribute to ZIKV-MAL pathogenesis, but several groups have demonstrated that ZIKV-MAL pathogenesis (32, 67, 68) aligns more closely with African lineage viruses.

Heat maps visualized NES values for two lineage infections (American [PR] and African [UG]), four GSEA pathways, and nine cell types at 7 dpi (Fig. 6E through H). The results demonstrate that UPR/ISR and apoptotic responses were downregulated in PR infections as compared to UG (Fig. 6E and F), while the antiviral and interferon responses were upregulated in PR relative to UG. These data advance a mechanistic understanding of American/Asian virus clearance due to interferon and antiviral influences, while African lineage viruses persist, activating damaging cellular stress responses.

ZIKV-MAL and ZIKV-UG infections were characterized by elevated ROS levels (Fig. 7A through C), linking oxidative stress to severe pathogenesis (58, 59, 69, 70). ROS imbalance has myriad potential effects (71), including cytochrome C release that activates the caspase apoptosis cascade or causes direct damage to DNA, proteins, or lipids. We interpret the results of the Trolox experiments (Fig. 7) as evidence that ROS imbalance is a critical mechanism of severe ZIKV pathogenesis. We note that a recent publication (72) concluded that ROS is not a significant pathogenesis determinant for ZIKV infections in cultured neuronal cells; however, only an American ZIKV (Brazil, PE243) was tested. In contrast, the work reported here includes ZIKV-MAL and African lineage ZIKV, where there is strong evidence for ROS imbalance (Fig. 7).

Our data demonstrate that, in the presence of Trolox, organoid ventricles retained cytoarchitecture during ZIKV-UG infection; however, the viral titer is also increased (Fig. 7). These results suggest that scavenging hydroxyl radicals improved host tolerance to ZIKV infection without addressing viral replication or titer. Drugs that modulate host responses without targeting virus replication are referred to as host-directed therapeutics (73–75). Trolox is not regularly used therapeutically, but dexamethasone has been used to improve COVID-19 patient outcomes (76), with associated delayed viral release. Related data demonstrated that ZIKV-infected IFNAR−/− mouse pups had significantly better morbidity/mortality outcomes than IFNAR+/+ mice, despite the fact that ZIKV replicated to nearly 100-fold higher levels in the IFNAR−/− mouse pups (77). It has been demonstrated that the pan-caspase inhibitor Z-VAD-FMK prevented ZIKV death of SOX+ cells (44). Our results parallel others (77, 78) in illustrating that pathogenesis is derived from both host responses and viral replication.

STAT2 is a transcription factor that is an integral component of the ISGF3 complex to induce ISG expression during interferon response. STAT2 protein loss could contribute indirectly to elevated stress responses by impairing the formation of the ISGF3 transcription complex that promotes interferon gene expression. Without ISGF3 signaling and interferon expression, antiviral ISG expression is predicted to be blunted (Fig. 5F), permitting ongoing viral replication that activates the UPR and ISG stress pathways, leading to ROS imbalance and cell death. The diminished STAT2 levels in ZIKV-MAL and ZIKV-UG infections (Fig. 6I) are consistent with Grant et al. (21), who reported that STAT2 is more stable in ZIKV-PR infections of Vero cells than in ZIKV-UG infections, where STAT2 was degraded by the proteasome. In dengue virus infections, STAT2 is marked for proteasomal degradation by a ubiquitin ligase (UBR4) that is recruited by the viral non-structural protein 5 (NS5) (79). Our data (Fig. 6I) could be explained by STAT2 proteolysis; however, an alternate interpretation is that, because both IFIT1 and STAT2 are themselves ISGs, general downregulation of ISG expression could cause varying states of the interferon response. A limitation of our work is that the mechanism of STAT2 loss was not defined and will require further study. A further limitation is that the organoid model system lacks recruited immune cells; however, organoids have been described as excellent models for studying viral pathogenesis (62, 63).

Asian/American lineage ZIKV infections can cause congenital Zika syndrome (80), which includes microcephaly. Although the Asian/American ZIKV studied here showed relatively mild pathogenesis compared to African lineage ZIKV, the ZIKV-PR strain does indeed cause organoid pathology (Fig. 4). Our data are consistent with Tripathi et al. (78), who concluded that African lineage ZIKV causes a short, severe pathogenesis associated with fetal demise in a mouse model, while the Asian/American ZIKV infections cause a prolonged period to pathogenesis, occasionally causing death.

Overall, our data suggest that ZIKV pathogenesis includes lineage-specific differential host response patterns that control expression of antiviral, apoptosis, stress response, and interferon genes. Side-by-side analysis in cerebral organoids derived from human embryonic stem cells revealed that African lineage and ancestral Asian ZIKV-MAL evade or diminish intrinsic antiviral control responses while driving maladaptive stress responses that culminate in virus persistence and tissue disruption caused by ROS imbalance. In contrast, contemporary Asian/American strains elicit antiviral programs that, through intrinsic innate immune responses, limit viral replication and spread. This comparative framework adds mechanistic detail toward understanding how closely related ZIKV strains produce distinct pathological outcomes, while further providing a foundation for future studies aimed at modulating host tolerance as an approach to managing viral infections.

## MATERIALS AND METHODS

### NGD 0.5× media

NGD 0.5× media was prepared as previously described (81).

### ZIKV stocks

Three African lineage strains (Uganda [MR766], Nigeria [IbH 30656], and Senegal [DAK AR 41524]), two Asian lineage strains (Malaysia P6-740) and Cambodia (FSS13020), as well as one American lineage strain isolated from Puerto Rico (PRVABC59) were obtained from BEI Resources. An American lineage strain isolated in Brazil (HS-2015-BA-01) was gifted by Dr. Mauro M. Teixeira, Albert Einstein College of Medicine. ZIKV strains were expanded in C6/36 mosquito cells in NGD 0.5× medium at 28°C.

### Plaque assay for viral titer

Viral titer was determined by standard plaque assay on BHK-21 cells (ATCC CCL10) (82). Throughout this paper, multiplicity of infection values (MOI) equal numbers of infectious viral particles, determined by plaque assay, added per cell.

### Phylogenetic analysis and comparison of amino acid sequences

Genome sequences were retrieved from GenBank (NIH). Chronograms were estimated using BEAST v2.2.129 with a GTR substitution model, discrete gamma distribution, and random local clock, as optimized for ZIKV phylogenetics.

### Human embryonic stem cell and neuronal progenitor cell maintenance and differentiation

WIBR3 human embryonic stem cell (hESCs) (NIH registry 10-0079) were maintained in feeder-free conditions with StemFlex media (Gibco) on Matrigel-coated (Corning) 6-well tissue culture dishes. For passaging, cells were detached as clumps using Versene Solution (Thermo) and replated at a ratio of 1:8–1:10.

### 2D cell culture ZIKV infection

Cells were infected at an MOI of one plaque-forming unit per cell for 1 h at 37°C, followed by media replacement. Samples were collected at 48 and 72 hpi and snap frozen for biochemical analysis.

### Cerebral organoid ZIKV infection and sample preparation

Cerebral organoids were generated from hESCs (83) using the STEMdiff Cerebral Organoid Kit (Stem Cell Technologies). Organoids (21 days post-embryoid body formation) were infected with 500 PFU per organoid (MOI ≈ 0.002 infectious virus particles per cell). Samples were collected at 7, 14, and 21 dpi. For protein and RNA analysis, organoids were washed and snap frozen. Organoids were fixed in 4% paraformaldehyde for immunostaining.

### Cryosectioning and immunofluorescence

Fixed organoids were cryoprotected in sucrose, embedded in OCT compound (Fisher), sectioned at 20 μm, and stained using antibodies against SOX2, cleaved caspase-3, ZIKV envelope, MAP2, and dsRNA (J2). Alexa Fluor-conjugated secondary antibodies and DAPI were used. Antibody and dilution details are in Table S2. Sections were mounted with ProLong Diamond.

### Fluoro-Jade C and MitoSOX staining

Degenerating neurons were detected using Fluoro-Jade C per manufacturer’s instructions (53). MitoSOX Red staining was performed on infected Vero cells at 24 hpi following the manufacturer’s protocol.

### Colocalization analysis

J2 and ZIKV envelope colocalization was quantified in Fiji (84) using the BIOP JACoP plugin with Otsu thresholding. Pearson’s and Manders’ coefficients were calculated, with significance assessed by Coste’s randomization.

### Trolox treatment

Trolox was prepared in DMSO and used at 100 μM. Following infection, organoids were cultured in Trolox- or vehicle-containing media until collection.

### Microscopy and image analysis

Images were acquired using Leica darkfield and Olympus FV1200 confocal microscopes. Quantification was performed in Fiji. Organoid size, nuclear counts, SOX2/J2 colocalization, and MAP2 area were measured as previously described (85), with StarDist (86) used for nuclear segmentation. Each field of view contained at least 250 nuclei.

### Immunoblotting

Samples were lysed in RIPA buffer, separated by SDS-PAGE, and transferred to PVDF membranes. Total protein was visualized with Direct Blue 71. Blots were probed with primary antibodies (Table S2), HRP-conjugated secondary antibodies, and developed by enhanced chemiluminescence.

### Bulk RNA isolation and sequencing

RNA was isolated using the RNeasy Mini Kit and sequenced by the MIT Genomics Core. RNA-seq data were used to quantify transcripts from the hg38 human assembly with the gencode version 46 basic annotation plus virus genomes (ZVMA: KX377336.1; ZVPR: KX087101.3; ZVUG: U963573.2), using the nf-core/maseq workflow revision 3.14.0 (87). Differential expression was performed with DESeq2, followed by GSEA and gene ontology analysis.

### Single-cell RNA sequencing and analysis

Organoids were dissociated at 7 dpi and processed using the 10× Genomics Chromium 3′ v3.1 platform. Reads were mapped with CellRanger to the GRCh38 reference genome plus ZIKV genomes and analyzed in Seurat. Cells were filtered, integrated, clustered, and annotated using established marker genes. Differential expression and GSEA were performed on a per-cell-type basis. Cell numbers are shown in Table S1.

### Statistical analyses

All statistics were performed using GraphPad Prism 9.0.2 software. Statistical significance was determined using two-way ANOVA with Tukey’s multiple comparisons test.

## Data Availability

The GitHub repository for the code used for bulk and single-cell RNAseq analyses can be found at https://github.com/KochInstitute-Bioinformatics/Gehrke_d7_infection.git. Bulk and single-cell RNA-Seq data are available from the Gene Expression Omnibus under accession number GSE29779.

## SUPPLEMENTAL MATERIAL

Fig S1 mbio.00863-26-s0001.tif Dendrogram and organoid formation timeline.

Fig. S2 mbio.00863-26-s0002.tif Schematic representation of a SOX2-positive organoid ventricle.

Fig. S3 mbio.00863-26-s0003.tif Colocalization.

Fig. S4 mbio.00863-26-s0004.tif Caspase staining.

Fig. S5 mbio.00863-26-s0005.tif Bubble plot.

Table S1 mbio.00863-26-s0006.tif Numbers of cells analyzed in single cell seq.

Table S2 mbio.00863-26-s0007.tif Details of antibodies used in the study.

ASM does not own the copyrights to Supplemental Material that may be linked to, or accessed through, an article. The authors have granted ASM a non-exclusive, world-wide license to publish the Supplemental Material files. Please contact the corresponding author directly for reuse.

## References

[1] Haddow AD, Schuh AJ, Yasuda CY, Kasper MR, Heang V, Huy R, Guzman H, Tesh RB, Weaver SC. 2012. Genetic characterization of Zika virus strains: geographic expansion of the Asian lineage. PLoS Negl Trop Dis 6:e1477. doi:10.1371/journal.pntd.0001477 22389730 PMC3289602

[2] Gutiérrez-Bugallo G, Piedra LA, Rodriguez M, Bisset JA, Lourenço-de-Oliveira R, Weaver SC, Vasilakis N, Vega-Rúa A. 2019. Vector-borne transmission and evolution of Zika virus. Nat Ecol Evol 3:561–569. doi:10.1038/s41559-019-0836-z 30886369 PMC8900209

[3] Weaver SC, Forrester NL, Liu J, Vasilakis N. 2021. Population bottlenecks and founder effects: implications for mosquito-borne arboviral emergence. Nat Rev Microbiol 19:184–195. doi:10.1038/s41579-020-00482-8 33432235 PMC7798019

[4] Faye O, Freire CCM, Iamarino A, Faye O, de Oliveira JVC, Diallo M, Zanotto PMA, Sall AA. 2014. Molecular evolution of Zika virus during its emergence in the 20^th^ century. PLoS Negl Trop Dis 8:e2636. doi:10.1371/journal.pntd.0002636 24421913 PMC3888466

[5] Cauchemez S, Besnard M, Bompard P, Dub T, Guillemette-Artur P, Eyrolle-Guignot D, Salje H, Van Kerkhove MD, Abadie V, Garel C, et al.. 2016. Association between Zika virus and microcephaly in French Polynesia, 2013–15: a retrospective study. Lancet 387:2125–2132. doi:10.1016/S0140-6736(16)00651-6 26993883 PMC4909533

[6] Cao-Lormeau V-M, Blake A, Mons S, Lastère S, Roche C, Vanhomwegen J, Dub T, Baudouin L, Teissier A, Larre P, et al.. 2016. Guillain-Barré syndrome outbreak associated with Zika virus infection in French Polynesia: a case-control study. Lancet 387:1531–1539. doi:10.1016/S0140-6736(16)00562-6 26948433 PMC5444521

[7] de Araújo TVB, Rodrigues LC, de Alencar Ximenes RA, de Barros Miranda-Filho D, Montarroyos UR, de Melo APL, Valongueiro S, de Albuquerque M de F, Souza WV, Braga C, et al., investigators from the Microcephaly Epidemic Research Group, Brazilian Ministry of Health, Pan American Health Organization, Instituto de Medicina Integral Professor Fernando Figueira, State Health Department of Pernambuco. 2016. Association between Zika virus infection and microcephaly in Brazil, January to May, 2016: preliminary report of a case-control study. Lancet Infect Dis 16:1356–1363. doi:10.1016/S1473-3099(16)30318-8 27641777 PMC7617035

[8] Mlakar J, Korva M, Tul N, Popović M, Poljšak-Prijatelj M, Mraz J, Kolenc M, Resman Rus K, Vesnaver Vipotnik T, Fabjan Vodušek V, et al.. 2016. Zika virus associated with microcephaly. N Engl J Med 374:951–958. doi:10.1056/NEJMoa1600651 26862926

[9] Moore CA, Staples JE, Dobyns WB, Pessoa A, Ventura CV, Fonseca E da, Ribeiro EM, Ventura LO, Neto NN, Arena JF, et al.. 2017. Characterizing the pattern of anomalies in congenital Zika syndrome for pediatric clinicians. JAMA Pediatr 171:288–295. doi:10.1001/jamapediatrics.2016.3982 27812690 PMC5561417

[10] Russo FB, Jungmann P, Beltrão-Braga PCB. 2017. Zika infection and the development of neurological defects. Cell Microbiol 19. doi:10.1111/cmi.12744 28370966

[11] Noronha L de, Zanluca C, Azevedo MLV, Luz KG, Santos CND dos. 2016. Zika virus damages the human placental barrier and presents marked fetal neurotropism. Mem Inst Oswaldo Cruz 111:287–293. doi:10.1590/0074-02760160085 27143490 PMC4878297

[12] Sheridan MA, Balaraman V, Schust DJ, Ezashi T, Roberts RM, Franz AWE. 2018. African and Asian strains of Zika virus differ in their ability to infect and lyse primitive human placental trophoblast. PLoS One 13:e0200086. doi:10.1371/journal.pone.0200086 29985932 PMC6037361

[13] Platt DJ, Smith AM, Arora N, Diamond MS, Coyne CB, Miner JJ. 2018. Zika virus-related neurotropic flaviviruses infect human placental explants and cause fetal demise in mice. Sci Transl Med 10:eaao7090. doi:10.1126/scitranslmed.aao7090 29386359 PMC6136894

[14] Li Y, Banerjee S, Wang Y, Goldstein SA, Dong B, Gaughan C, Silverman RH, Weiss SR. 2016. Activation of RNase L is dependent on OAS3 expression during infection with diverse human viruses. Proc Natl Acad Sci USA 113:2241–2246. doi:10.1073/pnas.1519657113 26858407 PMC4776461

[15] Liu Y, Wang M, Cheng A, Yang Q, Wu Y, Jia R, Liu M, Zhu D, Chen S, Zhang S, et al.. 2020. The role of host eIF2α in viral infection. Virol J 17:112. doi:10.1186/s12985-020-01362-6 32703221 PMC7376328

[16] Sadler AJ, Williams BRG. 2008. Interferon-inducible antiviral effectors. Nat Rev Immunol 8:559–568. doi:10.1038/nri2314 18575461 PMC2522268

[17] Daffis S, Szretter KJ, Schriewer J, Li J, Youn S, Errett J, Lin T-Y, Schneller S, Zust R, Dong H, et al.. 2010. 2′-O methylation of the viral mRNA cap evades host restriction by IFIT family members. Nature 468:452–456. doi:10.1038/nature09489 21085181 PMC3058805

[18] Lu AY, Gustin A, Newhouse D, Gale M Jr. 2023. Viral protein accumulation of Zika virus variants links with regulation of innate immunity for differential control of viral replication, spread, and response to interferon. J Virol 97:e0198222. doi:10.1128/jvi.01982-22 37162358 PMC10231147

[19] Loo Y-M, Owen DM, Li K, Erickson AK, Johnson CL, Fish PM, Carney DS, Wang T, Ishida H, Yoneyama M, et al.. 2006. Viral and therapeutic control of IFN-β promoter stimulator 1 during hepatitis C virus infection. Proc Natl Acad Sci USA 103:6001–6006. doi:10.1073/pnas.0601523103 16585524 PMC1458687

[20] Wu B, Hur S. 2013. Viral counterattack against the host innate immune system. Cell Res 23:735–736. doi:10.1038/cr.2013.32 23478300 PMC3674382

[21] Grant A, Ponia SS, Tripathi S, Balasubramaniam V, Miorin L, Sourisseau M, Schwarz MC, Sánchez-Seco MP, Evans MJ, Best SM, et al.. 2016. Zika virus targets human STAT2 to inhibit type I interferon signaling. Cell Host Microbe 19:882–890. doi:10.1016/j.chom.2016.05.009 27212660 PMC4900918

[22] White E. 1998. Regulation of apoptosis by adenovirus E1A and E1B oncogenes. Semin Virol 8:505–513. doi:10.1006/smvy.1998.0155

[23] Badu P, Baniulyte G, Sammons MA, Pager CT. 2024. Activation of ATF3 via the integrated stress response pathway regulates innate immune response to restrict Zika virus. J Virol 98:e0105524. doi:10.1128/jvi.01055-24 39212382 PMC11494902

[24] Turpin J, El Safadi D, Lebeau G, Krejbich M, Chatelain C, Desprès P, Viranaïcken W, Krejbich-Trotot P. 2022. Apoptosis during ZIKA virus infection: too soon or too late? Int J Mol Sci 23:1287. doi:10.3390/ijms23031287 35163212 PMC8835863

[25] Mufrrih M, Chen B, Chan S-W. 2021. Zika virus induces an atypical tripartite unfolded protein response with sustained sensor and transient effector activation and a blunted BiP response. mSphere 6:e0036121. doi:10.1128/mSphere.00361-21 34106769 PMC8265652

[26] Yuan L, Huang X-Y, Liu Z-Y, Zhang F, Zhu X-L, Yu J-Y, Ji X, Xu Y-P, Li G, Li C, et al.. 2017. A single mutation in the prM protein of Zika virus contributes to fetal microcephaly. Science 358:933–936. doi:10.1126/science.aam7120 28971967

[27] Nunes BTD, Fontes-Garfias CR, Shan C, Muruato AE, Nunes JGC, Burbano RMR, Vasconcelos PFC, Shi P-Y, Medeiros DBA. 2020. Zika structural genes determine the virulence of African and Asian lineages. Emerg Microbes Infect 9:1023–1033. doi:10.1080/22221751.2020.1753583 32419649 PMC8284969

[28] Shan C, Xia H, Haller SL, Azar SR, Liu Y, Liu J, Muruato AE, Chen R, Rossi SL, Wakamiya M, et al.. 2020. A Zika virus envelope mutation preceding the 2015 epidemic enhances virulence and fitness for transmission. Proc Natl Acad Sci USA 117:20190–20197. doi:10.1073/pnas.2005722117 32747564 PMC7443865

[29] Jaeger AS, Murrieta RA, Goren LR, Crooks CM, Moriarty RV, Weiler AM, Rybarczyk S, Semler MR, Huffman C, Mejia A, et al.. 2019. Zika viruses of African and Asian lineages cause fetal harm in a mouse model of vertical transmission. PLoS Negl Trop Dis 13:e0007343. doi:10.1371/journal.pntd.0007343 30995223 PMC6488094

[30] Pettersson J-O, Eldholm V, Seligman SJ, Lundkvist Å, Falconar AK, Gaunt MW, Musso D, Nougairède A, Charrel R, Gould EA, et al.. 2016. How did Zika virus emerge in the Pacific islands and Latin America? mBio 7:e01239-16. doi:10.1128/mBio.01239-16 27729507 PMC5061869

[31] Cugola FR, Fernandes IR, Russo FB, Freitas BC, Dias JLM, Guimarães KP, Benazzato C, Almeida N, Pignatari GC, Romero S, et al.. 2016. The Brazilian Zika virus strain causes birth defects in experimental models. Nature 534:267–271. doi:10.1038/nature18296 27279226 PMC4902174

[32] Shao Q, Herrlinger S, Zhu Y-N, Yang M, Goodfellow F, Stice SL, Qi X-P, Brindley MA, Chen J-F. 2017. The African Zika virus MR-766 is more virulent and causes more severe brain damage than current Asian lineage and dengue virus. Development 144:4114–4124. doi:10.1242/dev.156752 28993398 PMC5719247

[33] Anfasa F, Siegers JY, van der Kroeg M, Mumtaz N, Stalin Raj V, de Vrij FMS, Widagdo W, Gabriel G, Salinas S, Simonin Y, et al.. 2017. Phenotypic differences between Asian and African lineage Zika viruses in human neural progenitor cells. mSphere 2:e00292-17. doi:10.1128/mSphere.00292-17 28815211 PMC5555676

[34] Camp JG, Badsha F, Florio M, Kanton S, Gerber T, Wilsch-Bräuninger M, Lewitus E, Sykes A, Hevers W, Lancaster M, et al.. 2015. Human cerebral organoids recapitulate gene expression programs of fetal neocortex development. Proc Natl Acad Sci USA 112:15672–15677. doi:10.1073/pnas.1520760112 26644564 PMC4697386

[35] Lancaster MA, Renner M, Martin C-A, Wenzel D, Bicknell LS, Hurles ME, Homfray T, Penninger JM, Jackson AP, Knoblich JA. 2013. Cerebral organoids model human brain development and microcephaly. Nature 501:373–379. doi:10.1038/nature12517 23995685 PMC3817409

[36] Li Y, Muffat J, Omer A, Bosch I, Lancaster MA, Sur M, Gehrke L, Knoblich JA, Jaenisch R. 2017. Induction of expansion and folding in human cerebral organoids. Cell Stem Cell 20:385–396. doi:10.1016/j.stem.2016.11.017 28041895 PMC6461394

[37] Renner M, Lancaster MA, Bian S, Choi H, Ku T, Peer A, Chung K, Knoblich JA. 2017. Self‐organized developmental patterning and differentiation in cerebral organoids. EMBO J 36:1316–1329. doi:10.15252/embj.201694700 28283582 PMC5430225

[38] Qian X, Nguyen HN, Song MM, Hadiono C, Ogden SC, Hammack C, Yao B, Hamersky GR, Jacob F, Zhong C, et al.. 2016. Brain-region-specific organoids using mini-bioreactors for modeling ZIKV exposure. Cell 165:1238–1254. doi:10.1016/j.cell.2016.04.032 27118425 PMC4900885

[39] Salick MR, Wells MF, Eggan K, Kaykas A. 2017. Modelling Zika virus infection of the developing human brain in vitro using stem cell derived cerebral organoids. J Vis Exp:56404. doi:10.3791/56404 28994790 PMC5752258

[40] Ferent J, Zaidi D, Francis F. 2020. Extracellular control of radial glia proliferation and scaffolding during cortical development and pathology. Front Cell Dev Biol 8:578341. doi:10.3389/fcell.2020.578341 33178693 PMC7596222

[41] Di Lullo E, Kriegstein AR. 2017. The use of brain organoids to investigate neural development and disease. Nat Rev Neurosci 18:573–584. doi:10.1038/nrn.2017.107 28878372 PMC5667942

[42] Tang H, Hammack C, Ogden SC, Wen Z, Qian X, Li Y, Yao B, Shin J, Zhang F, Lee EM, et al.. 2016. Zika virus infects human cortical neural progenitors and attenuates their growth. Cell Stem Cell 18:587–590. doi:10.1016/j.stem.2016.02.016 26952870 PMC5299540

[43] Li C, Xu D, Ye Q, Hong S, Jiang Y, Liu X, Zhang N, Shi L, Qin C-F, Xu Z. 2016. Zika virus disrupts neural progenitor development and leads to microcephaly in mice. Cell Stem Cell 19:672. doi:10.1016/j.stem.2016.10.017 27814481

[44] Souza BSF, Sampaio GLA, Pereira CS, Campos GS, Sardi SI, Freitas LAR, Figueira CP, Paredes BD, Nonaka CKV, Azevedo CM, et al.. 2016. Zika virus infection induces mitosis abnormalities and apoptotic cell death of human neural progenitor cells. Sci Rep 6:39775. doi:10.1038/srep39775 28008958 PMC5180086

[45] Yang S, Gorshkov K, Lee EM, Xu M, Cheng Y-S, Sun N, Soheilian F, de Val N, Ming G, Song H, et al.. 2020. Zika virus-induced neuronal apoptosis via increased mitochondrial fragmentation. Front Microbiol 11:598203. doi:10.3389/fmicb.2020.598203 33424801 PMC7785723

[46] Komarasamy TV, Adnan NAA, James W, Balasubramaniam V. 2022. Zika virus neuropathogenesis: the different brain cells, host factors and mechanisms involved. Front Immunol 13:773191. doi:10.3389/fimmu.2022.773191 35371036 PMC8966389

[47] Daniel B, DeCoster MA. 2004. Quantification of sPLA_2_-induced early and late apoptosis changes in neuronal cell cultures using combined TUNEL and DAPI staining. Brain Res Brain Res Protoc 13:144–150. doi:10.1016/j.brainresprot.2004.04.001 15296851

[48] Eidet JR, Pasovic L, Maria R, Jackson CJ, Utheim TP. 2014. Objective assessment of changes in nuclear morphology and cell distribution following induction of apoptosis. Diagn Pathol 9:92. doi:10.1186/1746-1596-9-92 24885713 PMC4048047

[49] DeCoster MA. 2007. The nuclear area factor (NAF): a measure for cell apoptosis using microscopy and image analysis, p 378–384. *In* Modern research and educational topics in microscopy. Vol. 1.

[50] Helmy IM, Azim AMA. 2012. Efficacy of ImageJ in the assessment of apoptosis. Diagn Pathol 7:15. doi:10.1186/1746-1596-7-15 22309648 PMC3307432

[51] Schmued LC, Stowers CC, Scallet AC, Xu L. 2005. Fluoro-Jade C results in ultra high resolution and contrast labeling of degenerating neurons. Brain Res 1035:24–31. doi:10.1016/j.brainres.2004.11.054 15713273

[52] Boehmer D, Zanoni I. 2025. Interferons in health and disease. Cell 188:4480–4504. doi:10.1016/j.cell.2025.06.044 40845809 PMC12380125

[53] Bowen JR, Quicke KM, Maddur MS, O’Neal JT, McDonald CE, Fedorova NB, Puri V, Shabman RS, Pulendran B, Suthar MS. 2017. Zika virus antagonizes type I interferon responses during infection of human dendritic cells. PLoS Pathog 13:e1006164. doi:10.1371/journal.ppat.1006164 28152048 PMC5289613

[54] Smith JA. 2014. A new paradigm: innate immune sensing of viruses via the unfolded protein response. Front Microbiol 5:222. doi:10.3389/fmicb.2014.00222 24904537 PMC4032990

[55] Taniuchi S, Miyake M, Tsugawa K, Oyadomari M, Oyadomari S. 2016. Integrated stress response of vertebrates is regulated by four eIF2α kinases. Sci Rep 6:32886. doi:10.1038/srep32886 27633668 PMC5025754

[56] Morrison J, Aguirre S, Fernandez-Sesma A. 2012. Innate immunity evasion by Dengue virus. Viruses 4:397–413. doi:10.3390/v4030397 22590678 PMC3347034

[57] Chakraborti S, Chakraborti T, Chattopadhyay D, Shaha C. 2019. Oxidative stress in microbial diseases. Springer Nature.

[58] Almeida LT, Ferraz AC, da Silva Caetano CC, da Silva Menegatto MB, Dos Santos Pereira Andrade AC, Lima RLS, Camini FC, Pereira SH, da Silva Pereira KY, de Mello Silva B, et al.. 2020. Zika virus induces oxidative stress and decreases antioxidant enzyme activities in vitro and in vivo. Virus Res 286:198084. doi:10.1016/j.virusres.2020.198084 32622852

[59] Zhang Z, Rong L, Li Y-P. 2019. Flaviviridae viruses and oxidative stress: implications for viral pathogenesis. Oxid Med Cell Longev 2019:1–17. doi:10.1155/2019/1409582 PMC6720866 31531178

[60] Foo J, Bellot G, Pervaiz S, Alonso S. 2022. Mitochondria-mediated oxidative stress during viral infection. Trends Microbiol 30:679–692. doi:10.1016/j.tim.2021.12.011 35063304

[61] Giordano ME, Caricato R, Lionetto MG. 2020. Concentration dependence of the antioxidant and prooxidant activity of trolox in HeLa cells: involvement in the induction of apoptotic volume decrease. Antioxidants (Basel) 9:1058. doi:10.3390/antiox9111058 33137938 PMC7693461

[62] Vazquez C, Negatu SG, Bannerman CD, Sriram S, Ming G-L, Jurado KA. 2024. Antiviral immunity within neural stem cells distinguishes Enterovirus-D68 strain differences in forebrain organoids. J Neuroinflammation 21:288. doi:10.1186/s12974-024-03275-5 39501367 PMC11539839

[63] Rybak-Wolf A, Wyler E, Pentimalli TM, Legnini I, Oliveras Martinez A, Glažar P, Loewa A, Kim SJ, Kaufer BB, Woehler A, et al.. 2023. Modelling viral encephalitis caused by herpes simplex virus 1 infection in cerebral organoids. Nat Microbiol 8:1252–1266. doi:10.1038/s41564-023-01405-y 37349587 PMC10322700

[64] Veit EC, Salim MS, Jung MJ, Richardson RB, Boys IN, Quinlan M, Barrall EA, Bednarski E, Hamilton RE, Kikawa C, et al.. 2024. Evolution of STAT2 resistance to flavivirus NS5 occurred multiple times despite genetic constraints. Nat Commun 15:5426. doi:10.1038/s41467-024-49758-0 38926343 PMC11208600

[65] Breckenridge DG, Germain M, Mathai JP, Nguyen M, Shore GC. 2003. Regulation of apoptosis by endoplasmic reticulum pathways. Oncogene 22:8608–8618. doi:10.1038/sj.onc.1207108 14634622

[66] Wang L, Valderramos SG, Wu A, Ouyang S, Li C, Brasil P, Bonaldo M, Coates T, Nielsen-Saines K, Jiang T, et al.. 2016. From mosquitos to humans: genetic evolution of Zika virus. Cell Host Microbe 19:561–565. doi:10.1016/j.chom.2016.04.006 27091703 PMC5648540

[67] Simonin Y, Loustalot F, Desmetz C, Foulongne V, Constant O, Fournier-Wirth C, Leon F, Molès J-P, Goubaud A, Lemaitre J-M, et al.. 2016. Zika virus strains potentially display different infectious profiles in human neural cells. EBioMedicine 12:161–169. doi:10.1016/j.ebiom.2016.09.020 27688094 PMC5078617

[68] McDonald EM, Duggal NK, Brault AC. 2017. Pathogenesis and sexual transmission of Spondweni and Zika viruses. PLoS Negl Trop Dis 11:e0005990. doi:10.1371/journal.pntd.0005990 28985234 PMC5655359

[69] Ledur PF, Karmirian K, Pedrosa C da S, Souza LRQ, Assis-de-Lemos G, Martins TM, Ferreira J de C, de Azevedo Reis GF, Silva ES, Silva D, et al.. 2020. Zika virus infection leads to mitochondrial failure, oxidative stress and DNA damage in human iPSC-derived astrocytes. Sci Rep 10:1218. doi:10.1038/s41598-020-57914-x 31988337 PMC6985105

[70] Fialho EMS, Veras EM, de Jesus CM, Khouri R, Sousa PS, Ribeiro MRC, Costa LC, Gomes LN, Nascimento FRF, Silva AAM, et al.. 2023. Maternal immune response to ZIKV triggers high-inflammatory profile in congenital Zika syndrome. Viruses 15:220. doi:10.3390/v15010220 36680261 PMC9866085

[71] Popa-Wagner A, Mitran S, Sivanesan S, Chang E, Buga A-M. 2013. ROS and brain diseases: the good, the bad, and the ugly. Oxid Med Cell Longev 2013:963520. doi:10.1155/2013/963520 24381719 PMC3871919

[72] Mendonça-Vieira L de, Aníbal-Silva CE, Menezes-Neto A, Azevedo E de A, Zanluqui NG, Peron JPS, Franca R de O. 2021. Reactive oxygen species (ROS) are not a key determinant for Zika virus-induced apoptosis in SH-SY5Y neuroblastoma cells. Viruses 13:2111. doi:10.3390/v13112111 34834918 PMC8622630

[73] Kaufmann SHE, Dorhoi A, Hotchkiss RS, Bartenschlager R. 2018. Host-directed therapies for bacterial and viral infections. Nat Rev Drug Discov 17:35–56. doi:10.1038/nrd.2017.162 28935918 PMC7097079

[74] Wallis RS, O’Garra A, Sher A, Wack A. 2023. Host-directed immunotherapy of viral and bacterial infections: past, present and future. Nat Rev Immunol 23:121–133. doi:10.1038/s41577-022-00734-z 35672482 PMC9171745

[75] Forman HJ, Zhang H. 2021. Targeting oxidative stress in disease: promise and limitations of antioxidant therapy. Nat Rev Drug Discov 20:689–709. doi:10.1038/s41573-021-00233-1 34194012 PMC8243062

[76] Horby P, Lim WS, Emberson JR, Mafham M, Bell JL, Linsell L, Staplin N, Brightling C, Ustianowski A, Elmahi E, et al.. 2021. Dexamethasone in hospitalized patients with Covid-19. N Engl J Med 384:693–704. doi:10.1056/NEJMoa2021436 32678530 PMC7383595

[77] Yockey LJ, Jurado KA, Arora N, Millet A, Rakib T, Milano KM, Hastings AK, Fikrig E, Kong Y, Horvath TL, et al.. 2018. Type I interferons instigate fetal demise after Zika virus infection. Sci Immunol 3:eaao1680. doi:10.1126/sciimmunol.aao1680 29305462 PMC6049088

[78] Tripathi S, Balasubramaniam V, Brown JA, Mena I, Grant A, Bardina SV, Maringer K, Schwarz MC, Maestre AM, Sourisseau M, et al.. 2017. A novel Zika virus mouse model reveals strain specific differences in virus pathogenesis and host inflammatory immune responses. PLoS Pathog 13:e1006258. doi:10.1371/journal.ppat.1006258 28278235 PMC5373643

[79] Morrison J, Laurent-Rolle M, Maestre AM, Rajsbaum R, Pisanelli G, Simon V, Mulder LCF, Fernandez-Sesma A, García-Sastre A. 2013. Dengue virus co-opts UBR4 to degrade STAT2 and antagonize type I interferon signaling. PLoS Pathog 9:e1003265. doi:10.1371/journal.ppat.1003265 23555265 PMC3610674

[80] Krauer F, Riesen M, Reveiz L, Oladapo OT, Martínez-Vega R, Porgo TV, Haefliger A, Broutet NJ, Low N, WHO Zika Causality Working Group. 2017. Zika virus infection as a cause of congenital brain abnormalities and Guillain-Barré syndrome: systematic review. PLoS Med 14:e1002203. doi:10.1371/journal.pmed.1002203 28045901 PMC5207634

[81] Muffat J, Li Y, Omer A, Durbin A, Bosch I, Bakiasi G, Richards E, Meyer A, Gehrke L, Jaenisch R. 2018. Human induced pluripotent stem cell-derived glial cells and neural progenitors display divergent responses to Zika and dengue infections. Proc Natl Acad Sci USA 115:7117–7122. doi:10.1073/pnas.1719266115 29915057 PMC6142255

[82] Dulbecco R. 1952. Production of plaques in monolayer tissue cultures by single particles of an animal virus. Proc Natl Acad Sci USA 38:747–752. doi:10.1073/pnas.38.8.747 16589172 PMC1063645

[83] Lancaster MA, Knoblich JA. 2014. Generation of cerebral organoids from human pluripotent stem cells. Nat Protoc 9:2329–2340. doi:10.1038/nprot.2014.158 25188634 PMC4160653

[84] Schindelin J, Arganda-Carreras I, Frise E, Kaynig V, Longair M, Pietzsch T, Preibisch S, Rueden C, Saalfeld S, Schmid B, Tinevez J-Y, White DJ, Hartenstein V, Eliceiri K, Tomancak P, Cardona A. 2012. Fiji: an open-source platform for biological-image analysis. Nat Methods 9:676–682. doi:10.1038/nmeth.2019 22743772 PMC3855844

[85] Sundaramoorthy V, Green D, Locke K, O’Brien CM, Dearnley M, Bingham J. 2020. Novel role of SARM1 mediated axonal degeneration in the pathogenesis of rabies. PLoS Pathog 16:e1008343. doi:10.1371/journal.ppat.1008343 32069324 PMC7048299

[86] Schmidt U, Weigert M, Broaddus C, Myers G. 2018. Cell Detection with Star-Convex Polygons, p 265–273. In Medical Image Computing and Computer Assisted Intervention – MICCAI 2018. Springer International Publishing.

[87] Patel H, Ewels P, Manning J, Garcia MU, Peltzer A, Hammarén R, Botvinnik O, Talbot A, Sturm G, Bot N-C, et al.. 2024. nf-core/rnaseq: nf-core/rnaseq v3.18.0 - lithium Lynx. Zenodo. Available from: https://zenodo.org/records/14537300. Retrieved 23 Jan 2025.

