# Supplementary figures and images for: Zika virus infections of human stem cell-derived cerebral organoids reveal viral lineage-specific pathogenesis responses
Source: mBio. 2026 Jun 15;17(7):e00863-26. doi: 10.1128/mbio.00863-26 (PMC13343971; doi:10.1128/mbio.00863-26)

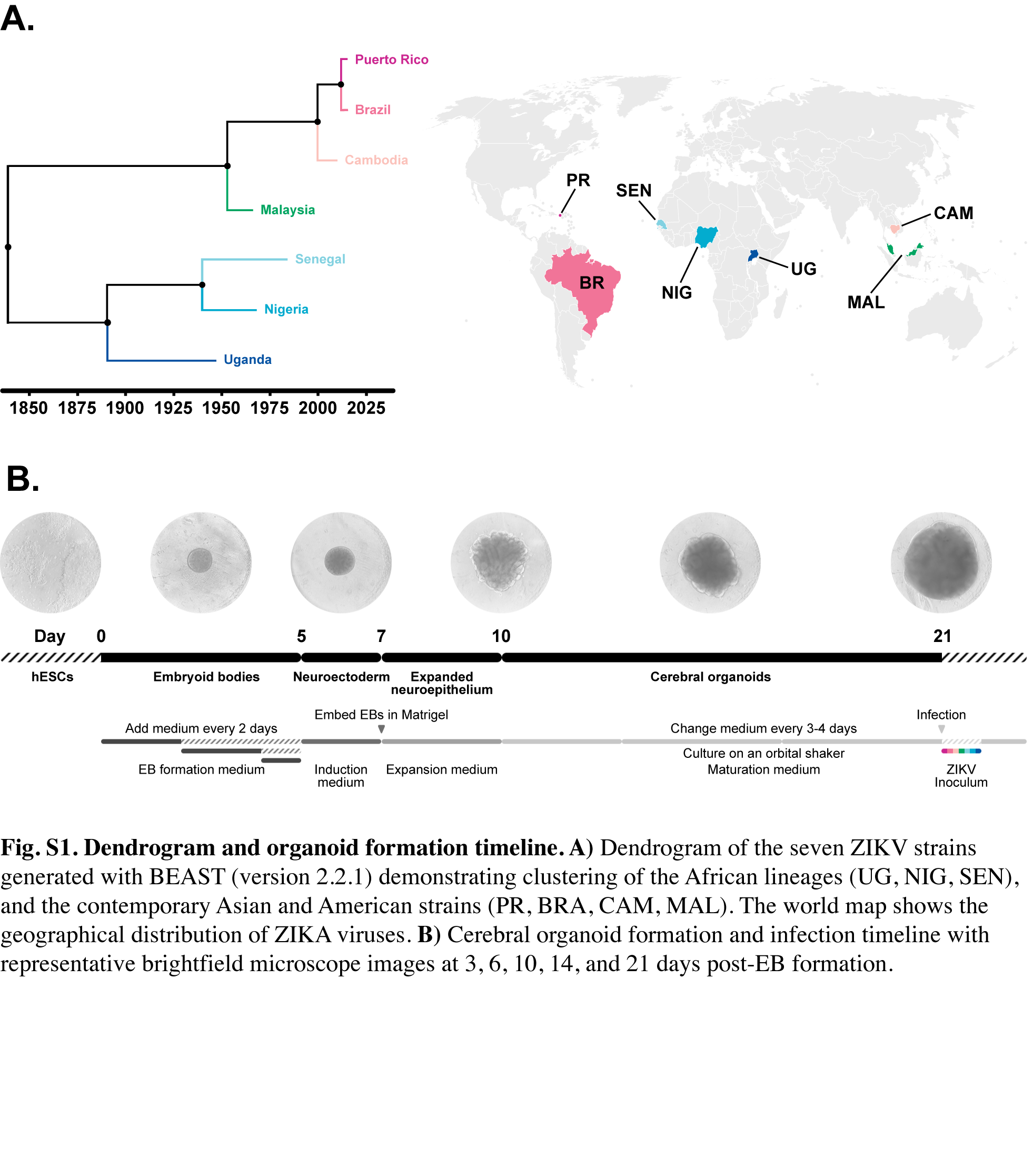

Supplement: Fig S1 — Dendrogram and organoid formation timeline. [file mbio.00863-26-s0001.tif]

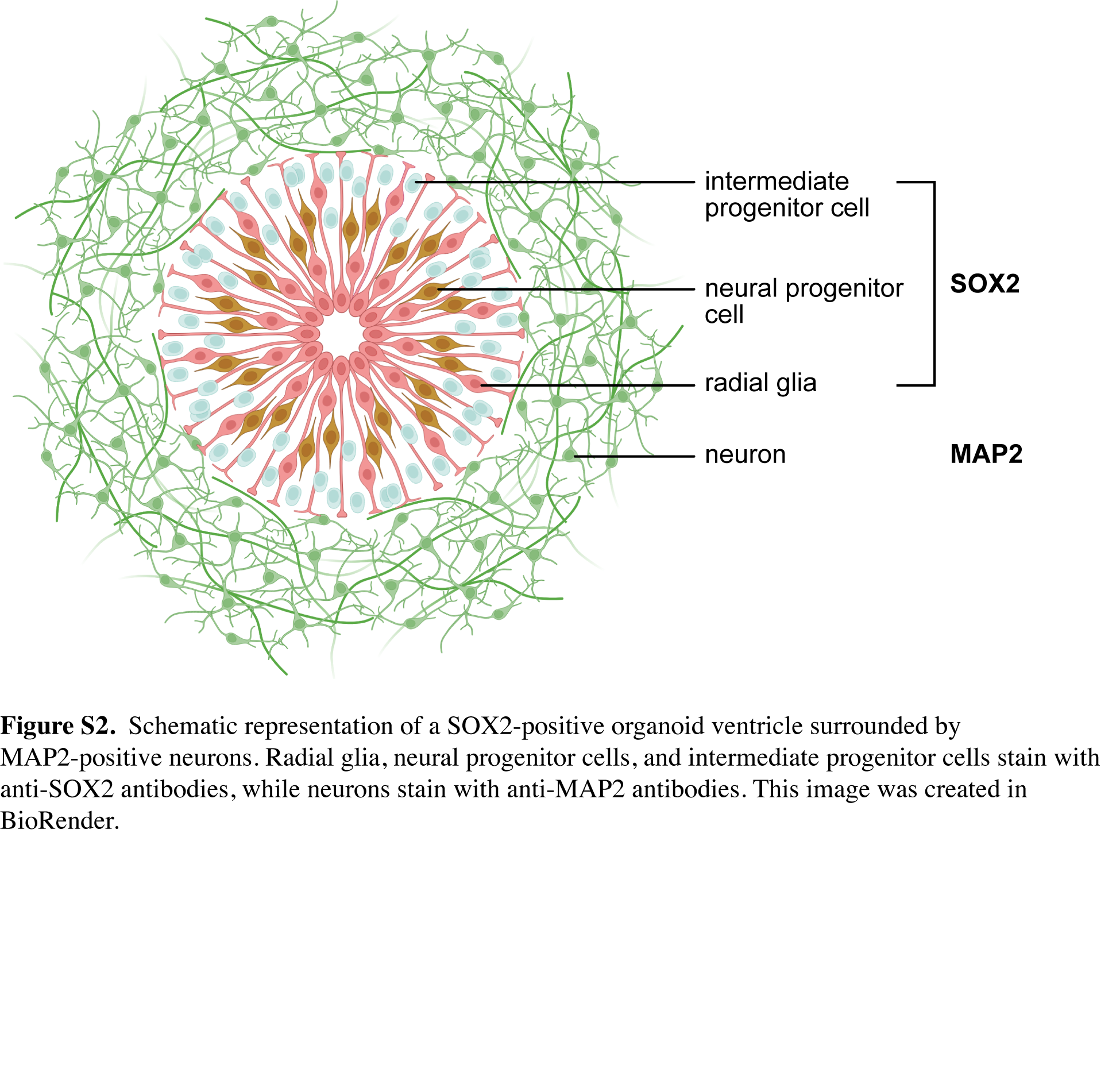

Supplement: Fig. S2 — Schematic representation of a SOX2-positive organoid ventricle. [file mbio.00863-26-s0002.tif]

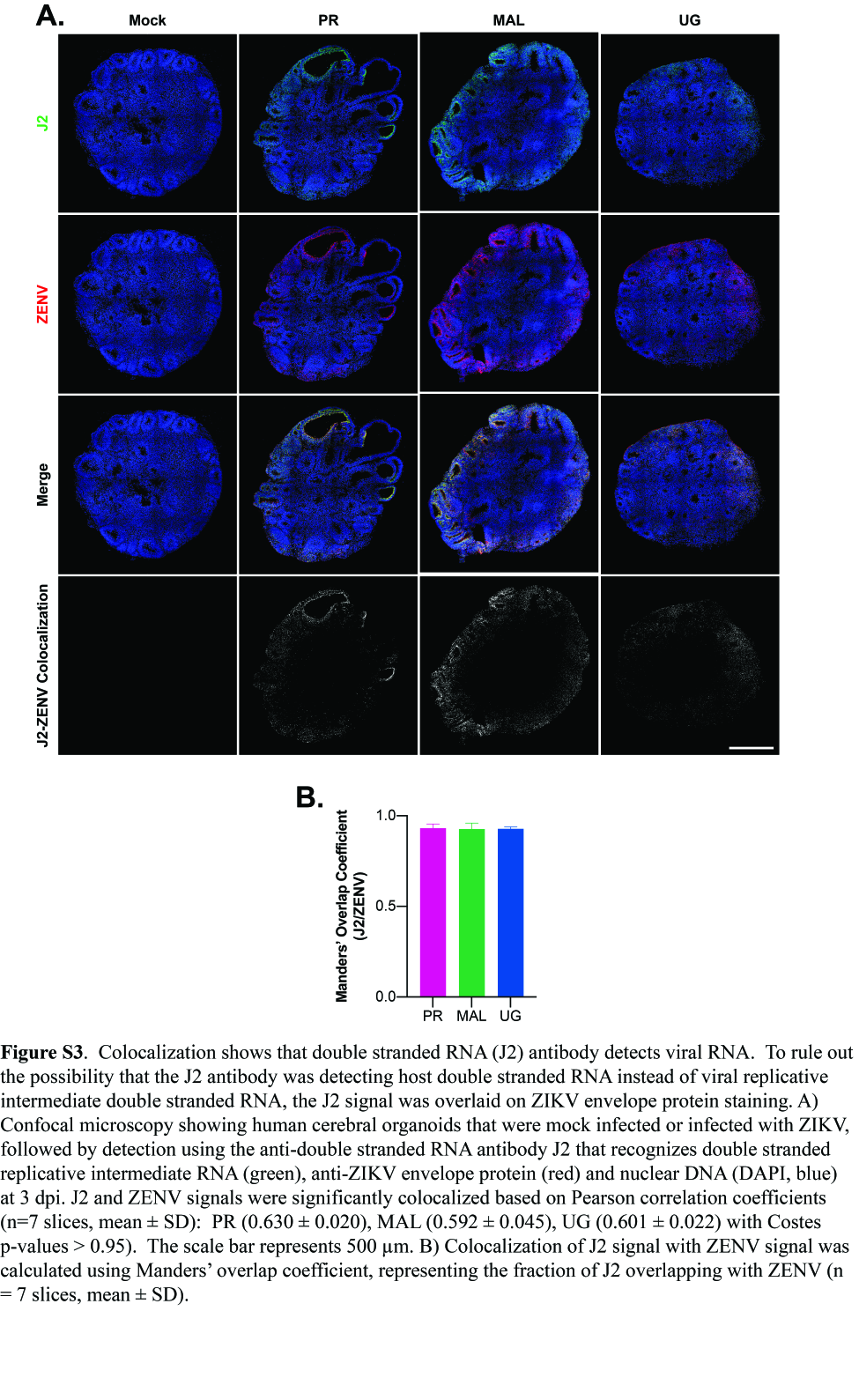

Supplement: Fig. S3 — Colocalization. [file mbio.00863-26-s0003.tif]

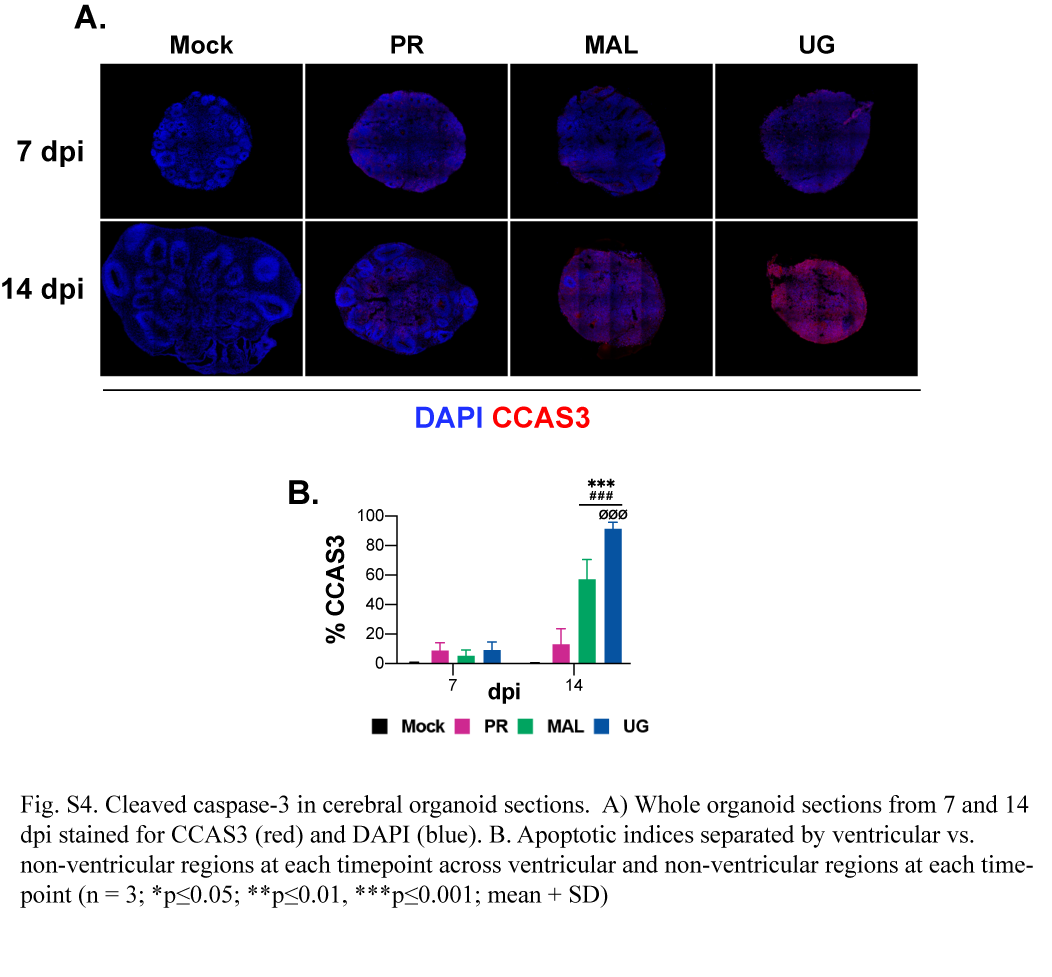

Supplement: Fig. S4 — Caspase staining. [file mbio.00863-26-s0004.tif]

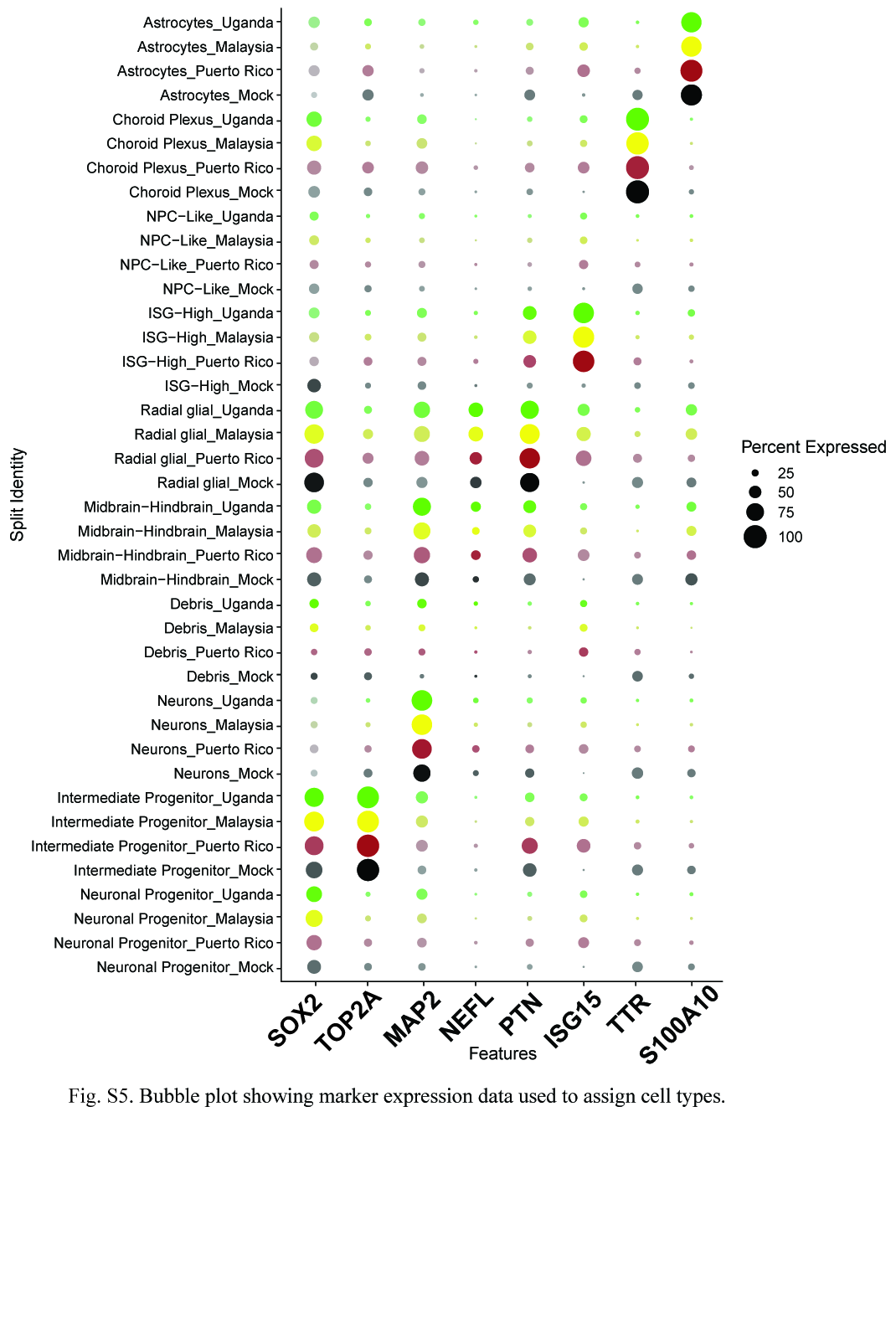

Supplement: Fig. S5 — Bubble plot. [file mbio.00863-26-s0005.tif]

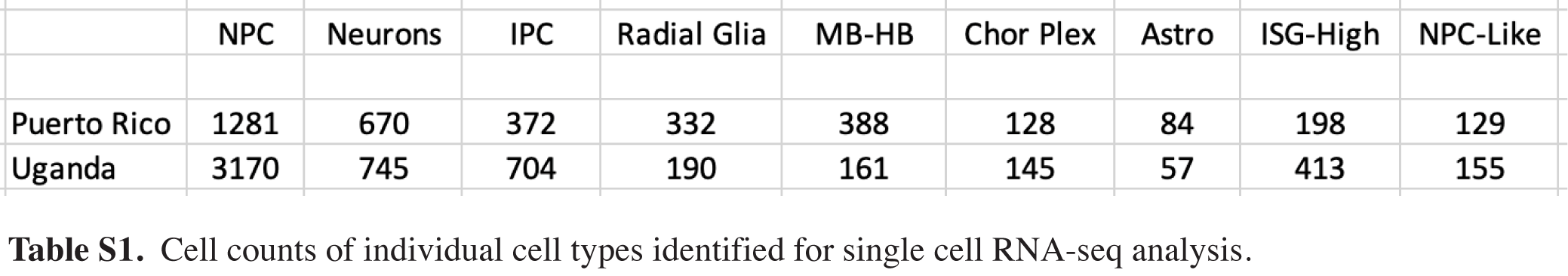

Supplement: Table S1 — Numbers of cells analyzed in single cell seq. [file mbio.00863-26-s0006.tif]

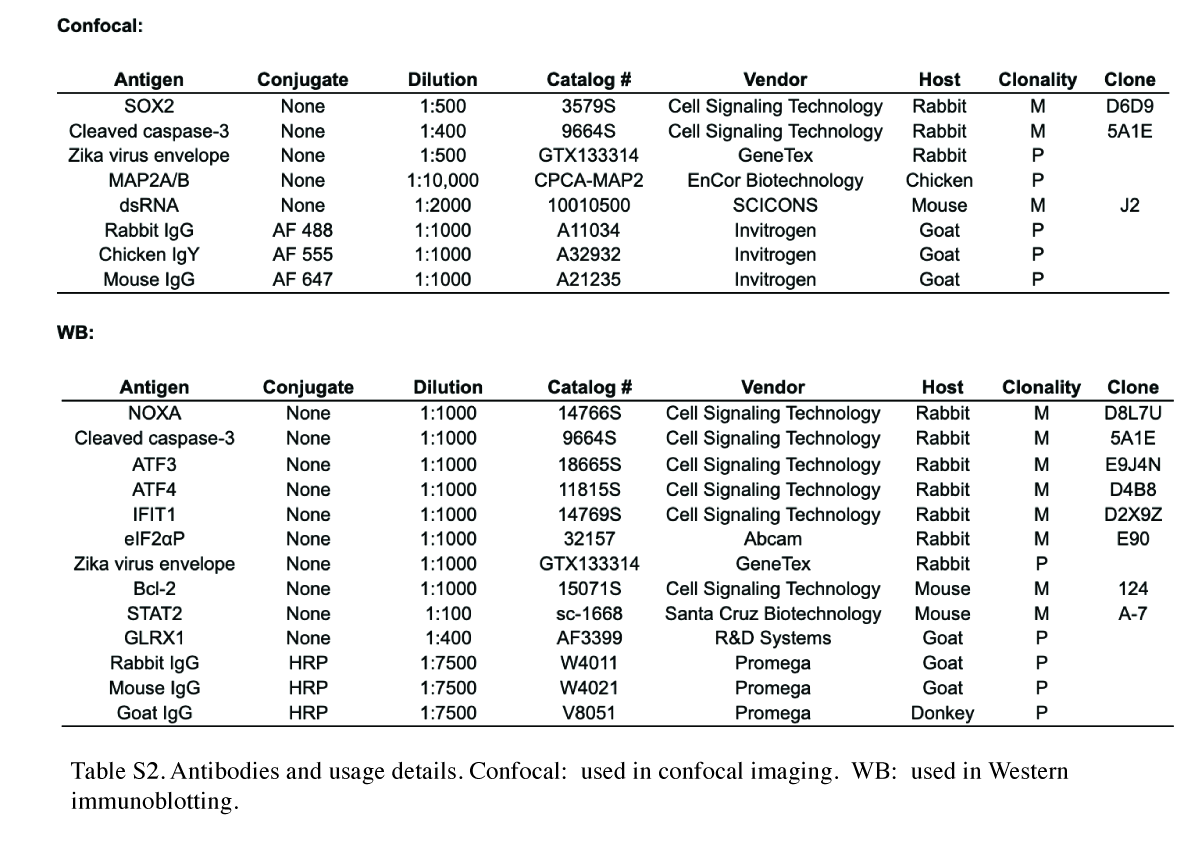

Supplement: Table S2 — Details of antibodies used in the study. [file mbio.00863-26-s0007.tif]
